# A common human missense mutation of vesicle coat protein SEC23B leads to growth restriction and chronic pancreatitis in mice

**DOI:** 10.1016/j.jbc.2021.101536

**Published:** 2021-12-24

**Authors:** Wei Wei, Zhigang Liu, Chao Zhang, Rami Khoriaty, Min Zhu, Bin Zhang

**Affiliations:** 1 Genomic Medicine Institute, Lerner Research Institute of Cleveland Clinic, Cleveland, Ohio, USA; 2 Department of Cardiovascular Surgery, Union Hospital, Tongji Medical College, Huazhong University of Science and Technology, Wuhan, China; 3 Departments of Internal Medicine, Cell and Developmental Biology and Rogel Cancer Center, University of Michigan, Ann Arbor, Michigan, USA; 4 Department of Pathology, Xinjiang Key Laboratory of Clinical Genetic Testing and Biomedical Information, Karamay Central Hospital, Karamay, China

**Keywords:** COPII, secretion, pancreas, growth hormone, ER stress, Larone syndrome, dyserythropoietic anemia, CDAII, congenital dyserythropoietic anemia type II, COPII, coat protein complex II, ER, endoplasmic reticulum, ES, embryonic stem, GH, growth hormone, GHR, growth hormone receptor, GTT, glucose tolerance test, IGF-1, insulin-like growth factor 1, IL, interleukin, JAK, Janus kinase, KI, knockin, *Neo*, neomycin, RBC, red blood cell, *Socs3*, suppressor of cytokine signaling-3, STAT, signal transducer and activator of transcription, TCA, trichloroacetic acid, TEM, transmission electron microscopy, TNF-α, tumor necrosis factor alpha, TUNEL, terminal deoxynucleotidyl transferase dUTP nick-end labeling, ZG, zymogen granule

## Abstract

SEC23B is one of two vertebrate paralogs of SEC23, a key component of the coat protein complex II vesicles. Complete deficiency of SEC23B in mice leads to perinatal death caused by massive degeneration of professional secretory tissues. However, functions of SEC23B in postnatal mice and outside professional secretory tissues are unclear. In this study, we generated a *Sec23b* KO mouse and a knockin (KI) mouse with the E109K mutation, the most common human mutation in congenital dyserythropoietic anemia type II patients. We found that E109K mutation led to decreases in SEC23B levels and protein mislocalization. However, *Sec23b*^*ki/ki*^ mice showed no obvious abnormalities. *Sec23b* hemizygosity (*Sec23b*^*ki/ko*^) was partially lethal, with only half of expected hemizygous mice surviving past weaning. Surviving *Sec23b*^*ki/ko*^ mice exhibited exocrine insufficiency, increased endoplasmic reticulum stress and apoptosis in the pancreas, and phenotypes consistent with chronic pancreatitis. *Sec23b*^*ki/ko*^ mice had mild to moderate anemia without other typical congenital dyserythropoietic anemia type II features, likely resulting from exocrine insufficiency. Moreover, *Sec23b*^*ki/ko*^ mice exhibited severe growth restriction accompanied by growth hormone (GH) insensitivity, reminiscent of Laron syndrome. Growth restriction is not associated with hepatocyte-specific *Sec23b* deletion, suggesting a nonliver origin of this phenotype. We propose that inflammation associated with chronic pancreatic deficiency may explain GH insensitivity in *Sec23b*^*ki/ko*^ mice. Our results reveal a genotype–phenotype correlation in SEC23B deficiency and indicate that pancreatic acinar is most sensitive to SEC23B deficiency in adult mice. The *Sec23b*^*ki/ko*^ mice provide a novel model of chronic pancreatitis and growth retardation with GH insensitivity.

Coat protein complex II (COPII) vesicles transport approximately 8000 mammalian proteins from the endoplasmic reticulum (ER) to the Golgi apparatus (1, 2, 3, 4). COPII is composed of five core components, including SAR1, SEC23, SEC24, SEC13, and SEC31 (5, 6). COPII vesicles assembly begins at distinct ER exit sites on the cytosolic surface marked by SEC16, where GDP-bound SAR1 is converted to GTP-bound SAR1 by the GTP exchange protein SEC12. SAR1-GTP recruits the SEC23–SEC24 heterodimer to form the inner layer of the COPII coat (7, 8, 9). The SAR1–SEC23–SEC24 prebudding complex further recruits the outer layer composed of SEC13–SEC31 heterotetramers, facilitating the budding of COPII vesicles from the surface of the ER (10, 11, 12).

In contrast to yeast, mammalian genomes contain two or more paralogs for most genes encoding COPII components (4, 13). Among these are two SEC23 paralogs, SEC23A and SEC23B, which share ∼85% amino acid sequence identity (4, 13). In humans, missense mutations in *SEC23A* lead to craniolenticulosutural dysplasia, characterized by craniofacial and skeletal abnormalities, in part because of collagen secretion defects (14, 15). We previously showed that mice with SEC23A deficiency exhibit midembryonic lethality, defective development of extraembryonic membranes, and neural tube opening in the midbrain, associated with secretion defects of multiple collagen types (16). Human mutations in *SEC23B* result in congenital dyserythropoietic anemia type II (CDAII) (17, 18), characterized by mild to moderate anemia, binucleated/multinucleated erythroblasts in the bone marrow, double membrane appearance in the red blood cell (RBC), and a faster shift and narrower band of RBC membrane protein band 3 on SDS-PAGE (19, 20). In CDAII patients, the majority of mutations are missense mutations, and no patients with two null mutations have been reported (21). E109K and R14W are two of the most frequent recurrent mutations in CDAII patients (22). Patients who are compound heterozygous with a null mutation and a missense mutation tend to have more severe anemia phenotypes than patients with two missense mutations (23), and hypomorphic mutations account for mild phenotypes of CDAII (24). In addition to causing CDAII, SEC23B mutations are also linked to Cowden's syndrome (25).

Despite the identification of genetic defects, the molecular mechanism of CDAII caused by SEC23B deficiency in humans remains unknown. We previously reported that mice with near complete deficiency for SEC23B were born with no apparent anemia phenotype, but died shortly after birth, with degeneration of professional secretory tissues, in particular degeneration of the pancreas (26). Pancreatic deficiency of SEC23B is the cause of perinatal lethality, and SEC23B is essential for the normal function of pancreatic acinar cells in adult mice (27, 28). Hematopoietic deficiency of SEC23B does not result in CDAII phenotype in adult mice (29). The lack of anemic phenotype in mice may be explained by differences in expression patterns SEC23 paralogs between mice and men. SEC23B is the dominant paralog in human erythropoietic cells, whereas the opposite is true for mice (29). A *Sec23a* coding sequence inserted into the murine *Sec23b* locus completely rescues the lethal SEC23B-deficient pancreatic phenotype, suggesting that SEC23A can substitute for SEC23B (30). However, it is unknown whether murine SEC23B is critical for functions outside professional secretory tissues. This question has been hampered by the lack of a global SEC23B-deficient mouse model that can survive beyond the first day after birth.

Here, we report the phenotype of SEC23B-deficient mice with the E109K missense mutation, which survive to adulthood. E109K hemizygous mice exhibited partial lethal phenotype, chronic pancreatitis, and severe postnatal growth retardation accompanied by growth hormone (GH) insensitivity. Our results suggest that SEC23B plays a critical role in mouse postnatal growth and pancreatic functions.

## Results

### The E109K mutation results in decreased SEC23B protein level and mislocalization of the mutant protein

**Figure 1:**
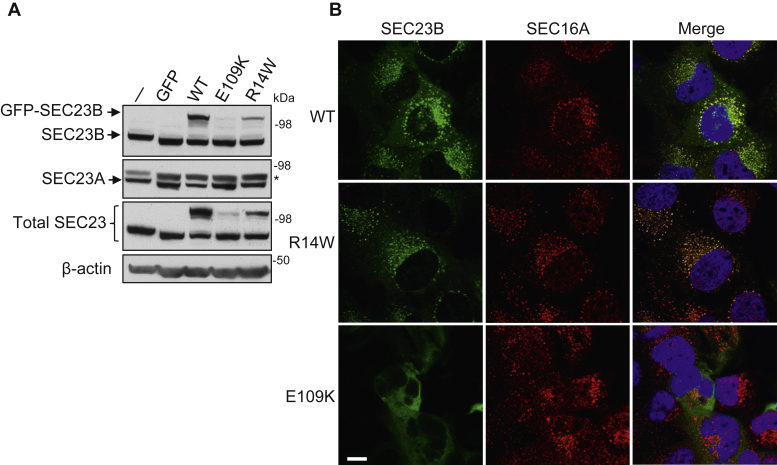
**Decreased protein levels and mislocation of SEC23B result from E109K missense mutation.***A,* immunoblotting analysis was performed to detect protein levels of endogenous and exogenous SEC23B, its endogenous paralog SEC23A, and total SEC23 in Nthy-ori 3-1 stable cell lines with GFP-tagged WT SEC23B, SEC23B^E109K^ mutant, or SEC23B^R14W^ mutant. *Asterisk* denotes a nonspecific band. Experiments were repeated three times. *B,* immunofluorescence staining of Nthy-ori 3-1 stable cell lines was performed to detect the intracellular localization of SEC23B. Nthy-ori 3-1 cells were stained with rabbit anti-Sec16A (*red*) for ER exit sites and DAPI (*blue*) for nuclei. Exogenous SEC23B-GFP fusion protein was stained *green*. The scale bar represents 10 μm. Experiments were repeated three times. DAPI, 4′,6-diamidino-2-phenylindole; ER, endoplasmic reticulum.

E109K is a founder mutation in the Italian population and also the most common mutation reported in CDAII patients (22). The R14W mutation is another founder mutation in the Italian population and the second most common mutation reported in CDAII patients (22). To study the impact of these mutations on SEC23B expression and function *in vitro*, we stably expressed human WT SEC23B, SEC23B^E109K^, and SEC23B^R14W^ mutants as GFP fusion proteins in Nthy-ori 3-1 cells, which are immortalized normal thyroid follicular epithelial cells as reported previously (25). We observed much lower expression of SEC23B^E109K^ and SEC23B^R14W^ than the WT protein (Fig. 1*A*). Expression of WT SEC23B, but not SEC23B mutants, led to a decrease in the SEC23A level (Fig. 1*A*). Both GFP-tagged WT SEC23B and SEC23B^R14W^ proteins colocalized with SEC16A, a marker for the ER exit sites (Fig. 1*B*). However, GFP-tagged SEC23B^E109K^ mutant failed to colocalize with SEC16A and became evenly distributed in the cytosol (Fig. 1*B*), suggesting that E109K and R14W mutants have different defects. Decreased expression level and mislocalization of the E109K mutant were also observed when SEC23B fusion proteins were transiently expressed in human embryonic kidney 293 cells (Fig. S1*A*). Treatment of cells with autophagy inhibitors chloroquine and 3-methyladenine, but not the proteasome inhibitor MG132, increased levels of SEC23B fusion proteins (Fig. S1*B*), suggesting that E109K and R14W mutants are primarily degraded through the autophagy pathway.

### Generation of mouse models with hemizygous E109K allele

**Figure 2:**
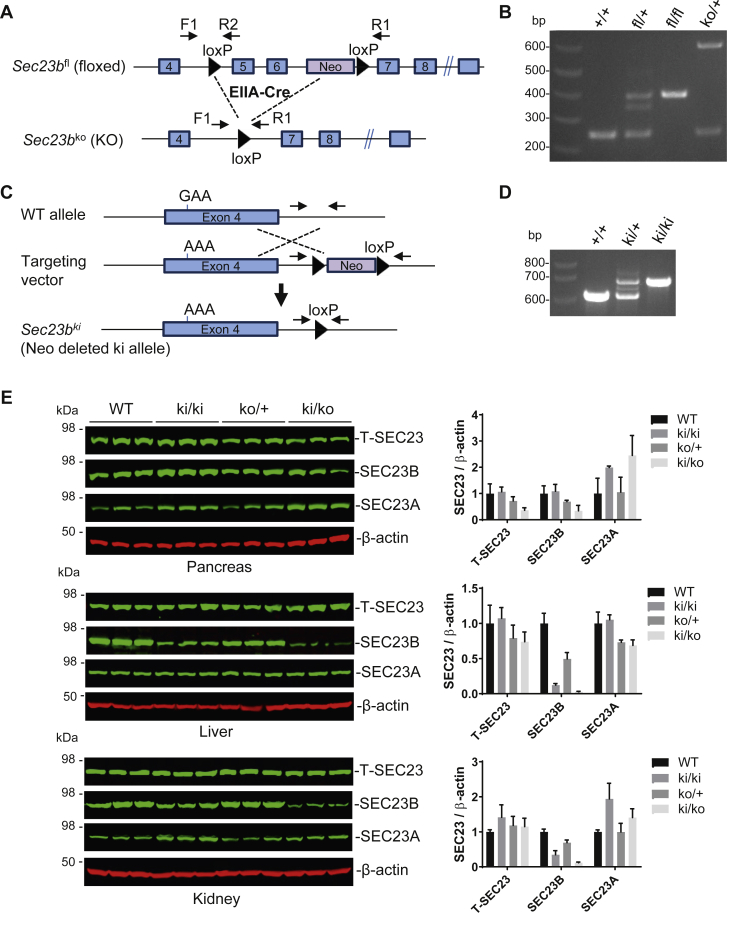
**Generation of *Sec23b* KO and knockin (KI) alleles.***A,* schematics of the *Sec23b* floxed allele and the conditional KO allele with deletion of exons 5 and 6. Positions of primer are indicated. *B,* a three-primer PCR assay distinguished floxed allele, KO allele, and WT allele. *C,* schematics of the KI allele with the E109K mutation within the exon 4. *D,* a two-primer PCR assay distinguished KI and WT alleles. Detailed diagrams on the generation of KO and KI alleles are shown in Figures S1 and S2. *E,* levels of SEC23 paralogs in SEC23B KO and E109K KI mouse models. Immunoblotting was performed to analyze total SEC23 (T-SEC23), SEC23B, SEC23A, and β-actin in pancreas, liver, and kidney from 8-month-old mice of the indicated genotypes. For each genotype, tissues from three mice were analyzed. IRDye 800CW-conjugated secondary antibodies were used in blots of SEC23 paralogs. IRDye 680RD-conjugated secondary antibodies were used in β-actin blots. Band intensities were quantified using the Odyssey Image Studio.

To study the impact of the E109K mutation *in vivo*, we generated two mouse lines. One is a *Sec23b* KO mouse line with the deletion of exons 5 and 6 (Figs. 2, *A* and *B* and S2). This mouse is distinct from the previously reported conditional gene-trap mouse that also deleted exons 5 and 6 (29). The second is a knockin (KI) mouse line carrying the SEC23B^E109K^ mutation (Figs. 2, *C* and *D* and S3). Heterozygous KO (*Sec23b*^*ko/+*^) mice exhibited normal survival (Fig. S4*A*), and no abnormalities were noted during routine necropsy, consistent with previously reported gene-trap mice (26, 29). Intercrosses of heterozygous KO mice produced homozygous KO (*Sec23b*^*ko/ko*^) pups that die shortly after birth with pancreas degeneration (Fig. S4, *B* and *C*), consistent with previously reported phenotype of SEC23B-deficient mice (26, 29). Heterozygous KI (*Sec23b*^*ki/+*^) mice exhibited normal survival, and no abnormalities were noted during routine necropsy. Intercrosses of heterozygous KI (*Sec23b*^*ki/+*^) mice produced homozygous KI (*Sec23b*^*ki/ki*^) mice. The KI allele contains a remnant loxP sequence in intron 4 after deletion of the neomycin (*Neo*) cassette by the cre recombinase (Fig. S3). To rule out the possibility that this intronic sequence may affect *Sec23b* expression and splicing, we measured mRNA levels of WT, *Sec23b*^*ki/+*^, and *Sec23b*^*ki/ki*^ mice by real-time RT–PCR, which could accurately detect a 50% reduction in the *Sec23b* mRNA level in *Sec23b*^*ko/+*^ mice (data not shown). No significant differences in *Sec23b* mRNA levels from liver, kidney, and pancreas were observed in all three mouse strains (Fig. S5A). RT–PCR using primers flanking the intron 4 junction detected no alternative splicing of *Sec23b* mRNA (Fig. S5B). Therefore, the remnant loxP site in intron 4 has no adverse effects on *Sec23b* transcription and RNA splicing.

### Expression levels of SEC23 paralogs in *Sec23b* KO and E109K KI mouse models

Intercrosses of heterozygous KO (*Sec23b*^*ko/+*^) mice with *Sec23b*^*ki/ki*^ or *Sec23b*^*ki/+*^ mice generated mice hemizygous for the SEC23B^E109K^ mutation (*Sec23b*^*ki/ko*^). Levels of SEC23A, SEC23B, and total SEC23 proteins from WT (*Sec23b*^*+/+*^), *Sec23b*^*ki/ki*^, *Sec23b*^*ko/+*^, and *Sec23b*^*ki/ko*^ mice were measured in pancreas, liver, and kidney lysates by immunoblotting. As shown in Figure 2*E*, SEC23B levels in *Sec23b*^*ki/ki*^ mice decreased in liver and kidney, but not in pancreas, compared with WT mice. SEC23B protein levels further decreased in all three organs in *Sec23b*^*ki/ko*^ mice compared with those of *Sec23b*^*ko/+*^ mice. Compensatory increase in SEC23A levels had been reported in tissues of *Sec23b* KO mice (26). Although a decrease in SEC23B level was not observed in pancreas of *Sec23b*^*ki/ko*^ mice, we found that SEC23A levels increased in both *Sec23b*^*ki/ki*^ and *Sec23b*^*ki/ko*^ mice compared with those of WT and *Sec23b*^*ko/+*^ mice (Fig. 2*E*), suggesting that SEC23B^E109K^ has functional defects, consistent with the *in vitro* results (Figs. 1 and S1). Increased SEC23A levels were sufficient to offset the decreased SEC23B, so that steady-state total SEC23 levels were comparable between WT and *Sec23b*^*ki/ki*^ mouse tissues tested. However, increased SEC23A levels were insufficient to offset decreased SEC23B levels in pancreas of *Sec23b*^*ki/ko*^ mice (Fig. 2*E*). Because of the lack of an antibody suitable for immunofluorescence, we were unable to determine the subcellular localization pattern of SEC23B^E109K^ in KO mouse cells. In WT mouse pancreas, the SEC23B level gradually decreased as mice age, whereas the SEC23A level gradually increased (Fig. S6). However, in *Sec23b*^*ki/ko*^ mouse pancreas, levels of both SEC23 paralogs remain relatively steady (Fig. S6).

### *Sec23b*^*E109K*^ hemizygosity leads to partial lethality and growth restrictions in the mouse

At the time of weaning, the number of *Sec23b*^*ki/ki*^ mice was slightly lower than expected, although the genotype distribution is not statistically different from the expected (*p* > 0.16, Table 1). At normal weaning time (day 21), the observed number of *Sec23b*^*ki/ko*^ mice was significantly lower than expected (*p* < 0.001, Table 2). To determine whether the loss of *Sec23b*^*ki/ko*^ pups occurred postnatally, we genotyped neonatal pups from the *Sec23b*^*ko/+*^ × *Sec23b*^*ki/ki*^ cross and found no significant loss of *Sec23b*^*ki/ko*^ neonates (*p* > 0.41, Table 2), suggesting that the loss of *Sec23b*^*ki/ko*^ pups primarily occurred postnatally.

**Table 1:** Genotype distribution of pups at weaning from intercrosses of *Sec23b*^*ki/+*^ mice

Genotype	+/+ (n)	ki/+ (n)	ki/ki (n)	*p*
Expected ratio	25	50	25	
Observed ratio (n = 195)	28.7 (56)	51.8 (101)	19.5 (38)	>0.16

**Table 2:** Genotype distribution of pups from intercrosses of *Sec23b*^*ki/ki*^ mice with *Sec23b*^ko/+^ mice

Genotype	ki/+ (n)	ki/ko (n)	*p*
Expected ratio	50%	50%	
P21 observed ratio (n = 190)	75.6% (144)	24.4% (46)	<0.001
P1 observed ratio (n = 98)	54% (53)	41% (45)	>0.4

**Figure 3:**
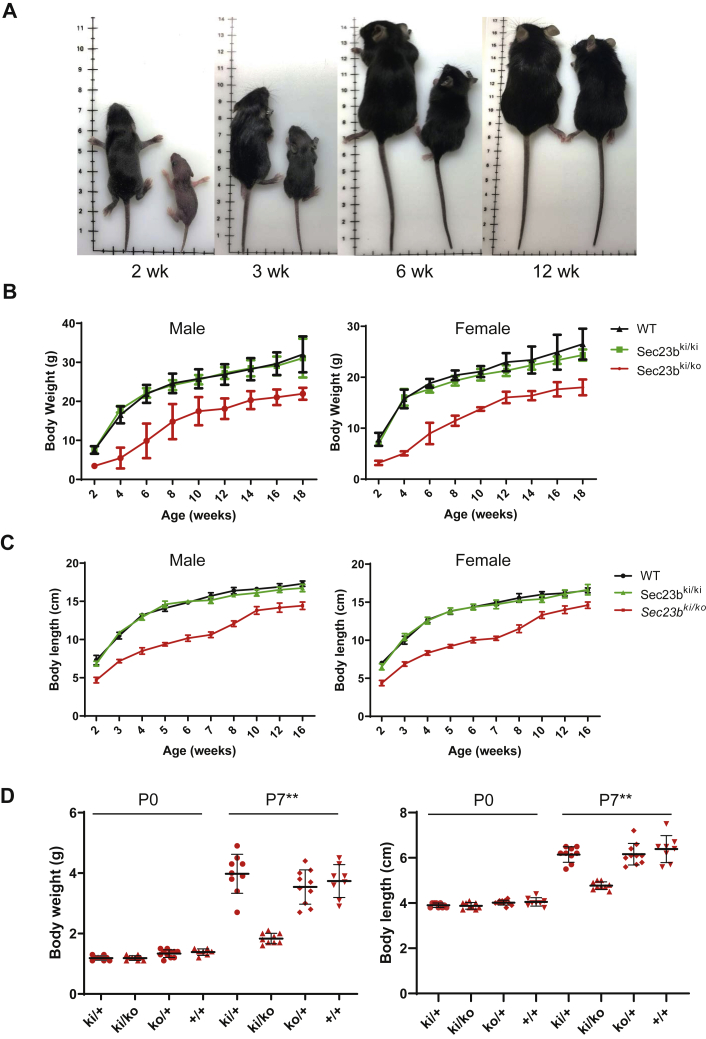
**Growth restriction in *Sec23B***^***ki/ko***^**mice.***A, Sec23b*^*ki/ko*^ mice (*right*) are consistently smaller than WT controls (*left*) of the same age. *B,* body weights of both male and female WT, *Sec23b*^*ki/ki*^, and *Sec23b*^*ki/ko*^ mice from 2 weeks of age to 18 weeks of age (data are mean ± SD, *p* < 0.05 at all time points). *C,* body lengths of both male and female WT, *Sec23b*^*ki/ki*^, and *Sec23b*^*ki/ko*^ mice from 2 weeks of age to 16 weeks of age (data are mean ± SD, *p* < 0.05 at all time points). *D,* body weights and lengths of *Sec23b*^*ki/+*^, and *Sec23b*^*ki/ko*^, *Sec23b*^*ko/+*^, and WT (*Sec23b*^*+/+*^) pups at P0 and P7.

We noticed that *Sec23b*^*ki/ko*^ mice appeared smaller in size compared with WT controls (Fig. 3*A*). In contrast, *Sec23b*^*ki/ki*^ and *Sec23b*^*ki/+*^ mice are indistinguishable from WT controls. Therefore, we monitored the body weight and body length of WT, *Sec23b*^*ki/ki*^, and *Sec23b*^*ki/ko*^ mice from the ages of 2 to 18 weeks. As shown in Figure 3*B*, body weights of both male and female *Sec23b*^*ki/ko*^ mice were consistently lower than those of WT or *Sec23b*^*ki/ki*^ mice throughout the study period. The differences among these mouse groups are especially striking within the first 6 weeks of life, with the body weights of *Sec23b*^*ki/ko*^ mice reaching only one-third of WT and *Sec23b*^*ki/ki*^ mice at week 4 (Fig. 3*B*). The differences narrowed between 8 and 12 weeks and remained relatively steady afterward. The trend of body length is very similar to that of body weight (Fig. 3*C*), which showed significantly decreased body lengths of *Sec23b*^*ki/ko*^ mice compared with the two other groups of mice. Even though an apparent growth spurt occurred in *Sec23b*^*ki/ko*^ mice from 7 to 10 weeks, both male and female adult *Sec23b*^*ki/ko*^ mice remained significantly shorter than WT and *Sec23b*^*ki/ki*^ mice. In contrast to *Sec23b*^*ki/ko*^ mice, the growth curve of *Sec23b*^*ki/ki*^ mice was similar to WT controls. To determine whether the size differences existed at birth, we further measured the weight and length of the offspring of the *Sec23b*^*ko/+*^ × *Sec23b*^*ki/ki*^ and *Sec23b*^*ko/+*^ × *Sec23b*^*ko/+*^ crosses as neonates (P0) and at 1 week after birth (P7). Although no significant differences were observed between WT, *Sec23b*^*ko/+*^, *Sec23b*^*ki/+*^, and *Sec23b*^*ki/ko*^ at P0, *Sec23b*^*ki/ko*^ pups had become significantly smaller at P7, both in weight and length (Fig. 3*D*), indicating that the growth restriction occurred postnatally.

### *Sec23b*^*E109K*^ hemizygous mice exhibit mild to moderate anemia without CDAII-like features

**Figure 4:**
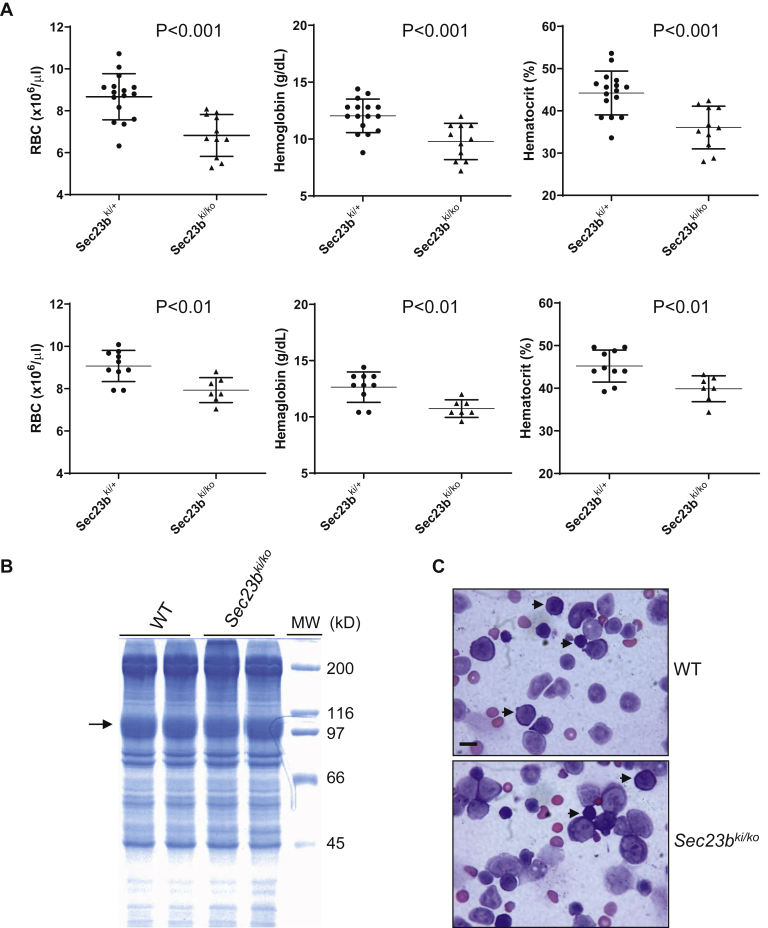
**Mild to moderate anemia in *Sec23b***^***ki/ko***^**mice without CDAII-like phenotype.***A,* RBC count, hemoglobin level, and hematocrit from 1-month-old (*top*) and 4-month-old (*bottom*) *Sec23b*^*ki/+*^ and *Sec23b*^*ki/ko*^ mice (data are mean ± SD). *B,* RBC ghosts were isolated from 1-month-old WT (n = 2) and *Sec23b*^*ki/ko*^ mice (n = 2) and separated by SDS-PAGE. Coomassie blue staining identified RBC membrane protein band 3 (*arrow*). *C,* Wright's stain of bone marrow smears of 4-month-old WT and *Sec23b*^*ki/ko*^ mice. *Arrows* indicate erythroblasts. The scale bar represents 10 μm. CDAII, congenital dyserythropoietic anemia type II; RBC, red blood cell.

Previous studies demonstrated that neither neonates of global SEC23B KO mice nor adult hematopoietic cell-specific SEC23B KO mice showed a CDAII-like phenotype (26, 29). We performed complete blood counts of 1-month-old and 4-month-old *Sec23b*^*ki/+*^ and *Sec23b*^*ki/ko*^ mice from the offspring of the *Sec23b*^*ko/+*^ × *Sec23b*^*ki/ki*^ cross. The RBC count, hemoglobin level, and hematocrit level were all significantly decreased in *Sec23b*^*ki/ko*^ mice, although the differences had narrowed from 1 month to 4 months (Fig. 4, *A* and *B*). Narrow band size and a shift in the mobility of membrane protein band 3 on SDS-PAGE are typical RBC abnormalities in CDAII patients. However, RBC from *Sec23b*^*ki/ko*^ mice exhibited no abnormalities in band 3 compared with WT RBC (Fig. 4*C*). A characteristic feature of bone marrow morphology of CDAII patients is the increased number of binuclear erythroblasts. However, no binucleated erythrocytes were observed in bone marrow smears of *Sec23b*^*ki/ko*^ mice (200 cells were observed for each mouse, two mice for each genotype) (Fig. 4*D*). Therefore, the anemia phenotype in *Sec23b*^*ki/ko*^ mice is distinct from the human CDAII phenotype.

### *Sec23b*^*E109K*^ hemizygosity results in pancreatic insufficiency

**Figure 5:**
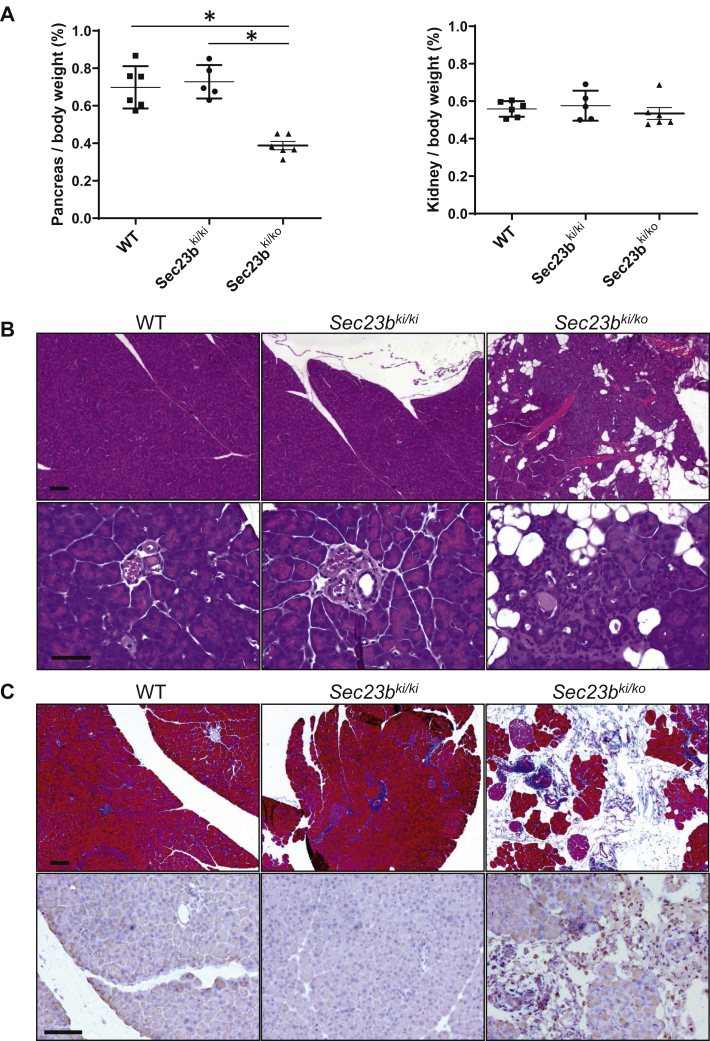
**Abnormal pancreas morphology of *Sec23b***^***ki/ko***^**mice.***A,* ratios of pancreas weight/body weight and kidney weight/body weight in WT, *Sec23b*^*ki/ki*^, and *Sec23b*^*ki/ko*^ mice (data are mean ± SD, ∗*p* < 0.05). *B,* H&E staining of pancreas from WT, *Sec23b*^*ki/ki*^, and *Sec23b*^*ki/ko*^ mice. The scale bars represent 100 μm (*top*) and 50 μm (*bottom*). *C, top panel,* Masson trichrome staining of pancreas from WT, *Sec23b*^*ki/ki*^, and *Sec23b*^*ki/ko*^ mice. The scale bar represents 100 μm. *Bottom panel,* immunohistochemistry staining of CD45 in pancreas from WT, *Sec23b*^*ki/ki*^, and *Sec23b*^*ki/ko*^ mice. Representative images from at least three biological replicates were shown. The scale bar represents 100 μm. All mice were 2 months old.

The ratio of pancreas weight/body weight is ∼40% lower for *Sec23b*^*ki/ko*^ mice than for WT and *Sec23b*^*ki/ki*^ mice, whereas the ratio of kidney weight/body weight is not significantly different between all groups (Fig. 5*A*). Histologic evaluation of pancreatic tissues of *Sec23b*^*ki/ko*^ mice demonstrated varying degrees of degeneration of acinar structure, ranging from near normal morphology to disruption of normal lobular structure because of multifocal degeneration of exocrine epithelia cells, prominence of fat tissue, and interstitial fibrosis (Figs. 5*B* and S7). Masson trichrome staining detected little blue-stained fibrous tissues in WT and *Sec23b*^*ki/ki*^ mouse pancreas (Fig. 5*C*). However, large amounts of blue collagen staining were readily observed in *Sec23b*^*ki/ko*^ mouse pancreas (Fig. 5*C*), which further revealed exocrine cell degeneration and pancreas fibrosis. In addition, we examined the inflammatory status of the pancreas by CD45 immunohistochemistry staining, which marks total white blood cells. Compared with scant CD45 staining in WT and *Sec23b*^*ki/ki*^ pancreas, a large number of white blood cells could be detected in *Sec23b*^*ki/ko*^ pancreas, and they were mainly distributed in the areas between gland tissues where increased fibrous and fat tissues are localized (Fig. 5*C*). We also measured the blood proinflammatory cytokines tumor necrosis factor alpha (TNF-α), interleukin (IL-1), and IL-6. TNFα and IL-1 levels were below detection limits, whereas the IL-6 level was significantly elevated in *Sec23b*^*ki/ko*^ mice compared with their WT littermates (Fig. S8), suggesting a chronic inflammation state in these mice. To investigate the onset of pancreatic phenotype, we evaluated histology of pancreatic tissues from littermates of *Sec23b*^*ki/ko*^ and *Sec23b*^*ki/+*^ neonates. H&E staining showed signs of minor degeneration of acinar cells and increased lymphocyte infiltration in *Sec23b*^*ki/ko*^ pancreas (Fig. S9). Therefore, pancreatic disruption of *Sec23b*^*ki/ko*^ mice was already present at birth. Complete deficiency in SEC23B also leads to degeneration of other professional secretory tissues (26). We compared the H&E staining of salivary glands of WT and *Sec23b*^*ki/ko*^ mice and found no obvious defects (Fig. S10), suggesting that pancreas is more sensitive to SEC23B deficiency.

**Figure 6:**
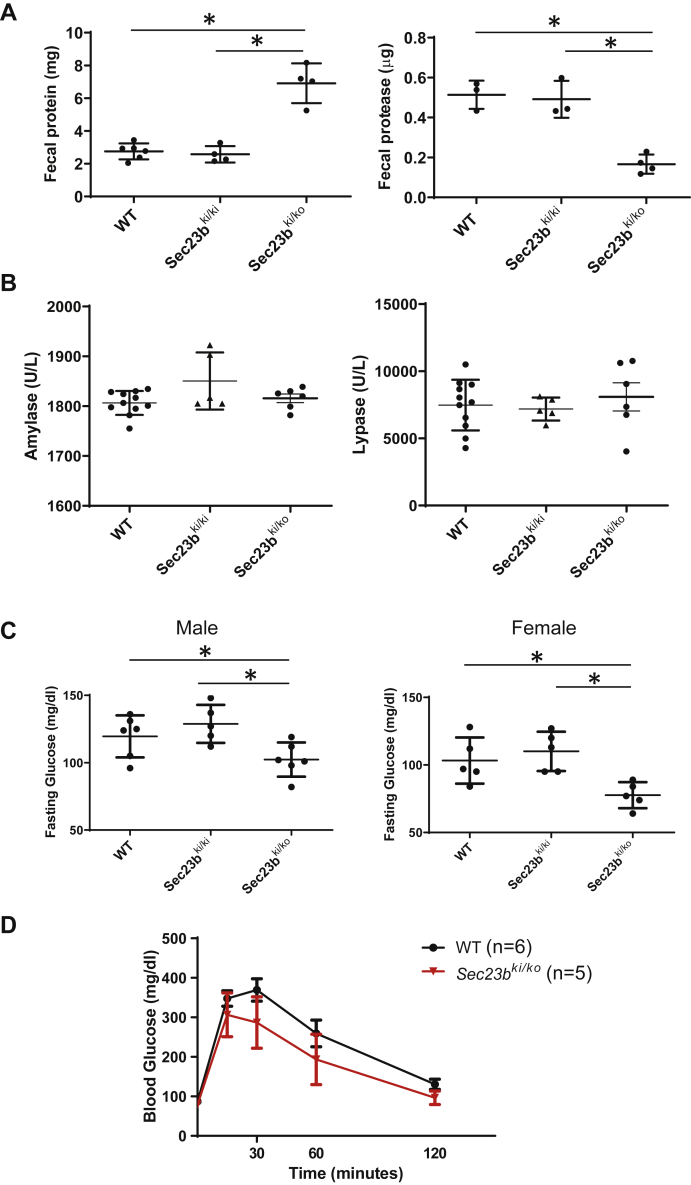
**Exocrine and endocrine functions in WT, *Sec23b***^***ki/ki***^**, and *Sec23b***^***ki/ko***^**mice.***A,* fecal protein and protease levels in WT, *Sec23b*^*ki/ki*^, and *Sec23b*^*ki/ko*^ mice (data are mean ± SD, *asterisks*: *p* < 0.05). *B,* serum amylase and lipase levels in WT, *Sec23b*^*ki/ki*^, and *Sec23b*^*ki/ko*^ mice (data are mean ± SD). *C,* blood glucose levels after 6 h starvation in both male and female mice of WT, *Sec23b*^*ki/ki*^, and *Sec23b*^*ki/ko*^ genotypes (data are mean ± SD, *asterisks*: *p* < 0.05). *C,* glucose tolerance test was conducted in male WT and *Sec23b*^*ki/ko*^ mice after overnight fasting. No differences in glucose levels were found at any time points between WT and *Sec23b*^*ki/ko*^ mice. All mice were 4 months old.

Next, we assessed the exocrine and endocrine functions of *Sec23b*^*ki/ko*^ mouse pancreas. To examine the exocrine function of pancreas, we collected dry or fresh stools and detected the amount of fecal protein and protease, which can indirectly assess pancreatic exocrine function (Fig. 6*A*). Over twofold more protein was detected in *Sec23b*^*ki/ko*^ mouse stools than in WT and *Sec23b*^*ki/ki*^ mouse stools (*p* < 0.05). Meanwhile, fecal protease level decreased over twofold in *Sec23b*^*ki/ko*^ mouse stools compared with WT and *Sec23b*^*ki/ki*^ mouse stools (Fig. 6*A*). However, serum amylase and lipase levels were not significantly different between WT, *Sec23b*^*ki/ko*^, and *Sec23b*^*ki/ki*^ mice (Fig. 6*B*). These experiments reveal significant deficiency in protein digestion in *Sec23b*^*ki/ko*^ mouse without significant accumulation of pancreatic enzymes in blood. To assess endocrine functions of the pancreas, we measured fasting glucose and performed glucose tolerance tests (GTTs) of WT and *Sec23b*^*ki/ko*^ mice. After 6 h of fasting, *Sec23b*^*ki/ko*^ mice exhibited mild to moderate hypoglycemia in both male and female mice (Fig. 6*C*). However, no significant difference was observed in GTT at any time points between WT and *Sec23b*^*ki/ko*^ mice (Fig. 6*D*). Immunostaining for insulin and glucagon demonstrated minor defects in islet architecture with a portion of alpha cells distributed within interior of the islet but no gross alteration in relative numbers of alpha and beta cells in *Sec23b*^*ki/ko*^ mouse islets (Fig. S11).

### *Sec23b*^*E109K*^ hemizygosity leads to ER stress and apoptosis of pancreatic cells

**Figure 7:**
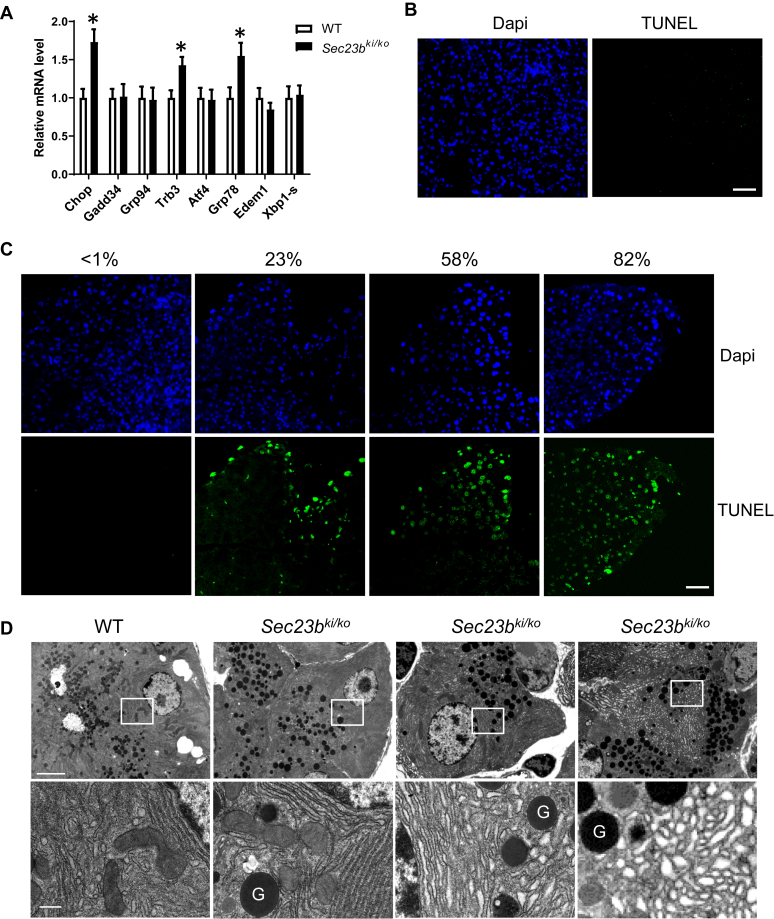
**ER stress, apoptosis, and ultrastructure of WT and *Sec23b***^***ki/ko***^**mouse pancreas.***A,* real-time RT–PCR quantification was conducted for expression of selected UPR genes in pancreas of 2-month-old WT (n = 6) and *Sec23b*^*ki/ko*^ (n = 6) mice. Data are mean ± SD, and *asterisks* indicate statistically significant differences between groups (*p* < 0.05). *B,* TUNEL staining of WT mouse pancreas at 2 months of age. Apoptotic cells were visualized in *green*, and nuclei are stained *blue* with DAPI. Less than 1% WT pancreatic cells were TUNEL positive. The scale bar represents 50 μm. *C,* increased apoptosis in the pancreas of *Sec23b*^*ki/ko*^ mice at 2 months of age. TUNEL staining detected different degrees of apoptosis in different regions of *Sec23b*^*ki/ko*^ pancreas, ranging from less than 1% to over 80% of TUNEL-positive cells. The scale bar represents 50 μm. *D,* variant degrees of ER abnormalities of pancreatic acinar cells from a *Sec23b*^*ki/ko*^ mouse compared with a WT mouse. *G,* zymogen granule. The scale bars represent 4 μm (*top*) and 0.5 μm (*bottom*). TEM images were representative of three mice from each genotype. DAPI, 4′,6-diamidino-2-phenylindole; ER, endoplasmic reticulum; TEM, transmission electron microscopy; TUNEL, terminal deoxynucleotidyl transferase dUTP nick-end labeling; UPR, unfolded protein response.

We first measured mRNA levels of genes in the ER stress pathway in WT and *Sec23b*^*ki/ko*^ mouse pancreas. Levels of heat shock protein family A member 5 (*Hspa5*; encoding glucose-regulated protein 78), DNA damage–inducible transcript 3 (*Ddit3*; encoding C/EBP homologous protein), and tribbles pseudokinase 3 (*Trib3*; encoding tribbles homolog 3) increased by 1.5-fold to 2.0-fold in *Sec23b*^*ki/ko*^ pancreas compared with those in WT pancreas (Fig. 7*A*). C/EBP homologous protein and tribbles homolog 3 are associated with apoptosis. Indeed, terminal deoxynucleotidyl transferase dUTP nick-end labeling (TUNEL) staining revealed different severities of apoptosis in different areas of *Sec23b*^*ki/ko*^ pancreas, ranging from less than 1% to over 80% of cells undergoing apoptosis (Fig. 7, *B* and *C*), corresponding to severities of exocrine degeneration (Fig. 5*B*). We next assessed pancreatic acinar cell ultrastructure using transmission electron microscopy (TEM). We observed various morphologies of acinar cells in *Sec23b*^*ki/ko*^ mouse pancreas (Fig. S12). There are cells with normal appearance of the ER and cells with mildly distended or disrupted ER cisternae, all with normal appearing zymogen granules (ZGs). These cells were often juxtaposed to cells at different stages of apoptosis. ZGs were still present in earlier-stage apoptotic cells and became absent in late-stage apoptotic cells (Fig. S12). The appearances of ER in zymogen-containing cells range between indistinguishable from WT cells and mildly distended ER with disruption of normal luminal structures (Fig. 7*D*). Acinar morphologies in *Sec23b*^*ki/ko*^ pancreas were in stark contrast to the previously reported *Sec23b*^*gt/gt*^ pancreas with complete SEC23B deficiency, in which acinar cells contained severely distended ER and were devoid of ZGs (26).

### GH insensitivity in *Sec23b*^*ki/ko*^ mice

**Figure 8:**
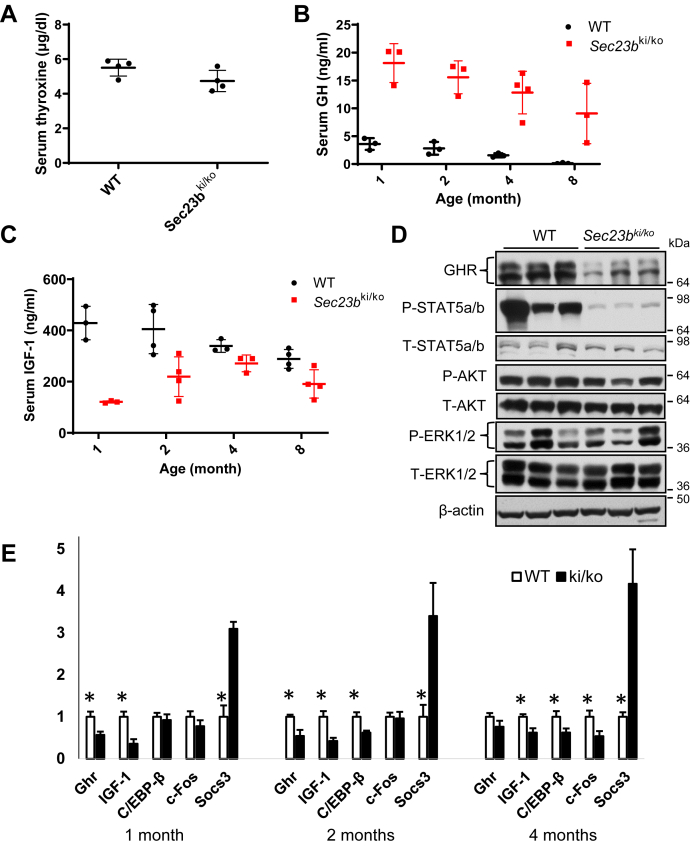
**Growth hormone (GH) insensitivity in *Sec23b***^***ki/ko***^**mice.***A,* comparison of serum thyroxine levels between WT and *Sec23b*^*ki/ko*^ mice at 2 months of age (data are mean ± SD, *p* > 0.05). *B,* comparison of circulating GH levels between WT and *Sec23b*^*ki/ko*^ mice from 1 to 8 months of age (data are mean ± SD, *p* < 0.05 at all time points). *C,* comparison of circulating IGF-1 levels between WT and *Sec23b*^*ki/ko*^ mice from 1 to 8 months of age (data are mean ± SD, *p* < 0.05 at all time points). *D,* GHR and downstream GHR signaling pathway components in liver lysates of WT and *Sec23b*^*ki/ko*^ mice at 2 months of age. P, phosphorylated T, total. *E,* real-time RT–PCR quantification of *Ghr* and select GHR target genes in WT and *Sec23b*^*ki/ko*^ liver at 1, 2, and 4 months of age. Data are mean ± SD. *Asterisks*: *p* < 0.05. IGF-1, insulin-like growth factor 1.

To understand the mechanism of growth restriction in *Sec23b*^*ki/ko*^ mice, we measured levels of two hormones important for growth, thyroxine and GH, in the serum of WT and *Sec23b*^*ki/ko*^ mice. Thyroxine levels were similar between WT and *Sec23b*^*ki/ko*^ groups (Fig. 8*A*). However, although steadily decreased as mice age, GH levels were markedly elevated in *Sec23b*^*ki/ko*^ mice compared with WT mice (Fig. 8*B*). Insulin-like growth factor 1 (IGF-1) is a major target of GH and mainly secreted by the liver. In contrast to GH, serum IGF-1 levels were lower in *Sec23b*^*ki/ko*^ mice than in WT mice, and the differences were most obvious during the first 2 months of age (Fig. 8*C*). Increased GH and decreased IGF-1 levels strongly suggest that *Sec23b*^*ki/ko*^ mice are resistant to GH. As a major target organ of GH, liver secretes circulating IGF-1 upon GH stimulation *via* the Janus kinase–signal transducer and activator of transcription (JAK–STAT) pathway. Therefore, we examined the GH receptor (GHR) and its downstream pathways, including JAK–STAT, PI3K–AKT, and mitogen-activated protein kinase pathways in mouse liver. As shown in Figure 8*D*, GHR level decreased significantly in *Sec23b*^*ki/ko*^ liver compared with WT liver. The intensity of phosphorylated STAT5a/b decreased to an extremely low level in *Sec23b*^*ki/ko*^ liver (Fig. 8*D*), whereas the total STAT5a/b remained unchanged. On the other hand, levels of phosphorylated AKT (PI3K–AKT pathway) and phosphorylated extracellular signal–regulated kinase 1/2 (mitogen-activated protein kinase pathway) in *Sec23b*^*ki/ko*^ liver appeared to decrease to a lesser extent (Fig. 8*D*). Next, we measured mRNA levels of *Ghr* and GHR-targeted genes in mouse liver by real-time RT–PCR. As shown in Figure 8*E*, the mRNA levels of *Ghr* and *Igf1* consistently decreased in 1-, 2-, and 4-month-old *Sec23b*^*ki/ko*^ mouse liver. The decrease was more prominent during the first 2 months. In contrast to *Ghr* and *Igf1*, the mRNA level of *Scos3*, which is a suppressor of GHR and the JAK–STAT pathway, was significantly higher in *Sec23b*^*ki/ko*^ liver. The mRNA levels of C/EBP-β and c-FOS decreased in certain age of *Sec23b*^*ki/ko*^ liver but not in all age groups. Therefore, different genes respond differently to the elevated GH level in *Sec23b*^*ki/ko*^ liver.

### Hepatic SEC23B deficiency is not the cause of growth restriction

**Figure 9:**
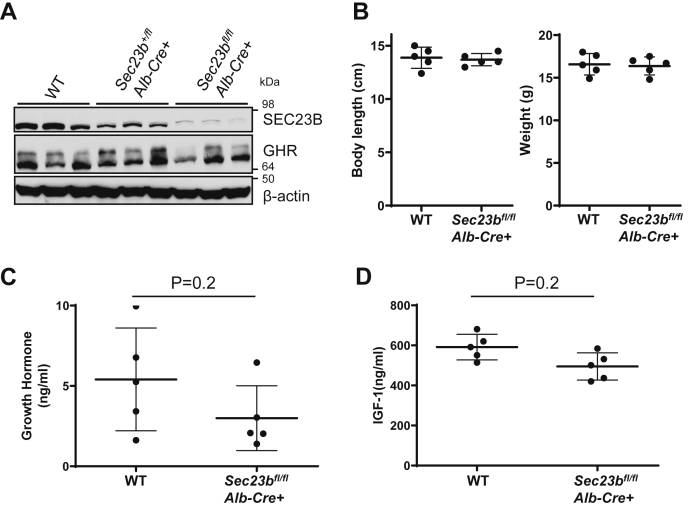
**No growth restriction and growth hormone (GH) insensitivity in *Sec23b***^***fl/fl***^***Alb-Cre* mice.***A,* immunoblotting analysis of SEC23B and GHR in liver lysates from 2-month-old WT, *Sec23b*^*+/fl*^*Alb-Cre*, and *Sec23b*^*fl/fl*^*Alb-Cre*^*+*^ mice (n = 3 for each genotype). *B,* body weights and lengths of WT and *Sec23b*^*fl/fl*^*Alb-Cre*^*+*^ male mice at 5 weeks of age. *C,* serum GH levels in WT and *Sec23b*^*fl/fl*^*Alb-Cre*^*+*^ mice. *D,* serum IGF-1 levels in WT and *Sec23b*^*fl/fl*^*Alb-Cre*^*+*^ mice. Both GH and IGF-1 levels were measured at 2 months of age by ELISA. All data showed no statistical difference between WT and *Sec23b*^*fl/fl*^*Alb-Cre*^*+*^ (data are mean ± SD, *p* > 0.05). GHR, growth hormone receptor; IGF-1, insulin-like growth factor 1.

To investigate whether SEC23B deficiency in liver is the main reason of growth restriction, we generated hepatocyte-specific *Sec23b* KO mice. To do this, we first crossed the *Sec23b*^*fl/+*^ mice with *Alb-Cre*^+^ mice that carry the Cre recombinase gene driven by the *Alb* promoter. The resulting *Sec23b*^*fl/+*^ *Alb-Cre*^+^ mice were then crossed with *Sec23b*^*fl/fl*^ mice to generate *Sec23b*^*fl/fl*^ *Alb-Cre*^+^ hepatocyte-specific *Sec23b* KO mice. As shown in Figure 9*A*, hepatocyte-specific deletion of *Sec23b* reduced SEC23B protein to an extremely low level in *Sec23b*^*fl/fl*^ *Alb-Cre*^+^ mouse liver. However, unlike *Sec23b*^*ki/ko*^ mice, we did not detect decreased GHR level in *Sec23b*^*fl/fl*^ *Alb-Cre*^+^ mouse liver (Fig. 9*A*). In contrast to *Sec23b*^*ki/ko*^ mice, *Sec23b*^*fl/fl*^ *Alb-Cre*^+^ mice appeared grossly normal and did not exhibit growth restriction (Fig. 9*B*). Serum GH and IGF-1 levels in *Sec23b*^*fl/fl*^ *Alb-Cre*^+^ mice were similar to those of WT mice (Fig. 9, *C* and *D*). Therefore, GH resistance of *Sec23b*^*ki/ko*^ liver was not caused by hepatic SEC23B deficiency.

## Discussion

Previous studies showed that mice with complete SEC23B deficiency die perinatally because of extensive degeneration of professional secretory tissues, especially pancreas (26, 29). Our new *Sec23b* KO strain (*Sec23b*^*ko/ko*^) confirmed this observation. Subsequent study on mice with pancreas-specific deletion of *Sec23b* showed that the pancreatic defect is the main cause of perinatal lethality (28). Tamoxifen-inducible and pancreatic acinar cell–specific *Sec23b* deletion further demonstrated that SEC23B is required for normal function of pancreatic acinar cells in adult mice (27). However, tamoxifen-inducible and pancreatic acinar cell–specific *Sec23b* deletion is only suitable for short-term study of the impact of SEC23B on the function of the pancreas. We report the characterization of mice with homozygous and hemizygous E109K mutation of *Sec23b*. The human counterpart of this mutation is the most common missense mutation identified in CDAII patients. Our *in vitro* studies showed that the E109K mutation both decreases the steady-state expression of SEC23B in cells and affects it localization to ER exit sites. Mice with homozygous SEC23B^E109K^ mutant alleles (*Sec23b*^*ki/ki*^) are grossly normal, suggesting that SEC23B carrying the E109K mutation still retains partial function. In addition, the SEC23A level is elevated in *Sec23b*^*ki/ki*^ mice, largely compensating for the decreases in SEC23B expression and function. Further reducing the gene dosage dramatically altered the survivability and phenotype, as postnatal death and pancreatic insufficiency are associated with hemizygous SEC23B^E109K^ mutation, but not with homozygous SEC23B^E109K^ mutation. Surviving *Sec23b*^*ki/ko*^ mice also exhibit severe growth restriction. Since the number of neonatal *Sec23b*^*ki/ko*^ pups is not significantly different from the expected number, and the weight and body length of *Sec23b*^*ki/ko*^ neonates are normal, the E109K hemizygosity is compatible with embryonic development. Thus, the partial lethality and growth restriction are the results of failure to thrive after birth. The first 3 weeks of life are most critical, as half of *Sec23b*^*ki/ko*^ mice died during this period. Body weight and length differences between WT and *Sec23b*^*ki/ko*^ mice narrowed as mice age, coinciding with the decrease of SEC23B level in WT pancreas, suggesting relatively decreased reliance on SEC23B over time. In humans, phenotype severity also correlates with the residual SEC23B activity, as more severe anemic phenotype was observed in CDAII patients with a missense allele and a null allele (23).

Our study indicates that pancreas remains the most susceptible organ to SEC23B deficiency postnatally, consistent with the expression of SEC23B as the dominant paralog in murine pancreas (26, 28). Hemizygous SEC23B^E109K^ mutation leads to chronic changes in the pancreas, which have not been found in other SEC23B-deficient mice, including degeneration and inflammation of the pancreatic tissue, increased fat deposition, and interstitial fibrosis of the pancreas accompanied by significant infiltration of white blood cells. These changes in *Sec23b*^*ki/ko*^ pancreas meet the criteria of morphologic changes in chronic pancreatitis. Mice with tamoxifen-inducible and acinar cell–specific SEC23B deficiency exhibit pancreatic cell loss but without an effect on viability of the mice or significant evidence of inflammation (27). Besides histologic changes in the pancreas, chronic pancreatitis also leads to exocrine and endocrine defects in human. One of the major phenotypes of exocrine deficiency is malabsorption of lipid and protein, which can result in malnutrition in humans. In our study, deficiency in protein absorption was indirectly detected in *Sec23b*^*ki/ko*^ mice, suggesting specific disruption of protease transport, such as chymotrypsinogen and carboxypeptidase, in pancreatic acinar cells. There is evidence that different enzymes move through the early secretory pathway to ZGs at different rates and thus may be differently affected by SEC23B deficiency (31). We did not observe increases in amylase and lipase levels in *Sec23b*^*ki/ko*^ mice. In human chronic pancreatitis, blood amylase and lipase levels are often not elevated or only slightly elevated, except during acute attacks.

In contrast to all the reported SEC23B-deficient mice so far, *Sec23b*^*ki/ko*^ mice did exhibit a mild to moderate anemia phenotype. However, this anemia is not accompanied by other characteristic features of CDAII, including increased binucleated erythrocytes and hypoglycosylated band 3 in mature RBC membranes. Lack of CDAII features is most likely because of SEC23A as the dominant paralog expressed in the hematopoietic cells (26, 29), in contrast to humans in which SEC23A is lost prior to SEC23B during normal human terminal erythroid differentiation (32). The anemia phenotype is likely caused by undernutrition because of malabsorption as a result of pancreatic insufficiency. Similar to previous studies, no major disruption in islet structure was observed in *Sec23b*^*ki/ko*^ mice, and intact islets can be observed after the loss of surrounding acinar cells. Interestingly, we found hypoglycemia in *Sec23b*^*ki/ko*^ mice after fasting, whereas hyperglycemia is usually observed in chronic pancreatitis patients. At the same time, there was no significant difference in GTT, suggesting no severe destruction of islet β-cell functions. Low storage of glycogen caused by malnutrition could explain the fasting hypoglycemia in *Sec23b*^*ki/ko*^ mice. Thus, SEC23B appears to be more important for exocrine than endocrine functions of the mouse pancreas.

Blockage of this secretory pathway can result in accumulation of exocrine pancreatic enzymes in the ER, which further induces ER stress. In both humans and mice, ER stress has been found in association with pancreatitis (33, 34, 35, 36). In humans, variants in procarboxypepidase A1 and mutations in chymotrypsinogen C were associated with both chronic pancreatitis and ER stress (33, 36). In addition, alterations in the activity of trypsinogen cause hereditary pancreatitis resulting from ER stress, such as mutations in the *PRSS1* and *SPINK1* genes (34, 37). In our study, we detected ER stress and increased apoptosis in *Sec23b*^*ki/ko*^ pancreas. Acinar cells with different levels of ER distension and varying numbers of ZGs were observed by TEM, including acinar cells at different stages of apoptosis. This is in contrast to severe ER distension and complete lack of ZGs in total SEC23B-deficient mice (26). This observation suggests that acinar cells with *Sec23b*^E109K^ hemizygosity can function for a period, allowing for new pancreas tissues to regenerate (38) before succumbing to apoptosis induced by continued ER stress, and may explain the relatively mild pancreas degeneration. Therefore, ER stress caused by SEC23B deficiency may trigger chronic pancreatitis in *Sec23b*^*ki/ko*^ mice.

To investigate the underlying reasons accounting for the growth restriction in *Sec23b*^*ki/ko*^ mice, we unexpectedly found increased GH and decreased IGF-1 levels in *Sec23b*^*ki/ko*^ mice, which strongly suggested GH insensitivity in these mice. GH plays a pivotal role in linear growth by binding to its widely expressed transmembrane receptor GHR, which subsequently activates downstream pathways and modulates the expression of its target genes. We further confirmed GH resistance by demonstrating impaired GHR signaling in *Sec23b*^*ki/ko*^ liver. Several conditions have been found to be related to GH resistance, such as loss-of-function mutations in the GH gene, undernutrition, imbalance of the endocrine system, and inflammation (39, 40). In humans, a disease called Laron syndrome is caused by defects in GHR (41, 42). Laron syndrome patients have similar phenotype to *Sec23b*^*ki/ko*^ mice in growth restriction, increased GH, and decreased serum IGF-1 levels. Considering the function of SEC23B in intracellular protein transport, we asked whether SEC23B deficiency results in a defect in transporting GHR. Therefore, we generated hepatic *Sec23b* conditional KO mice. Liver-specific deletion of GHR resulted in more than 90% suppression of serum IGF-1 and an over threefold increase in GH level (43). However, we did not observe significant differences in IGF-1 and GH levels between WT and *Sec23b*^*−/−*^ *Alb-cre*+ mice, suggesting normal GHR transport in SEC23B-deficient hepatocytes.

GH insensitivity can occur in inflammatory states in both clinical and experimental settings (44, 45, 46, 47, 48). Previous studies demonstrated that exogenous proinflammatory cytokines, such as IL-6, TNF-α, and IL-1β, inhibit GH signaling *in vitro* and *in vivo* (49, 50, 51). As discussed previously, chronic pancreatitis and the resulting undernutrition in *Sec23b*^*ki/ko*^ mice might be the major cause of GH resistance by generating a whole-body chronic inflammatory state. Consistent with this hypothesis, we observed elevated IL-6 levels in *Sec23b*^*ki/ko*^ mice. In addition, we found increased suppressor of cytokine signaling-3 (*Socs3*) mRNA level in *Sec23b*^*ki/ko*^ liver. SOCS3 is a major mediator of inflammation-induced GH resistance in liver (44, 50, 52). Upon inflammatory cytokine stimulation, IL-6 in particular, SOCS3 downregulates the JAK2–STAT5 pathway in several ways such as inhibiting *Ghr* transcriptional activity and inhibiting GH activation of the signal transducer STAT5b by binding with the membrane-proximal tyrosine residues of GHR (44, 53). Consistent with the well-known effect of SOCS3 on JAK2–STAT5, we detected a dramatic decrease in phosphorylated STAT5 levels in *Sec23b*^*ki/ko*^ liver. It is also well known that proinflammatory cytokines are able to reduce GHR mRNA and protein levels both *in vivo* and *in vitro* (45, 49, 54, 55), consistent with the decreased GHR level observed in *Sec23b*^*ki/ko*^ liver. Therefore, proinflammatory cytokines triggered by chronic pancreatitis could be one of the main reasons for the GH resistance observed in our mice.

## Experimental procedures

### Generation of *Sec23b* conditional KO mice

The *Sec23b* KO mice were produced from a vector that was designed to delete exons 5 and 6 of *Sec23b* in C57BL/6 embryonic stem (ES) cells. The 5′ homology arm (∼4.6 kb containing exons 3 and 4), the 3′ homology arm (∼5.0 kb containing exons 5–9), and the center piece (∼1.0 kb containing exons 5 and 6) were amplified by PCR using C57BL/6 bacterial artificial chromosome DNA as a template and cloned into the targeting vector LoxFtNwCD. The targeting construct was linearized using NotI and electroporated into C57BL/6 ES cells. The targeted allele was identified by Southern blot. Mice with germline transmission of the targeting allele (*Sec23b*^*fl/+*^ mice) were continually crossed with C57BL/6J mice. Global KO mice were generated by crossing *Sec23b*^*fl/+*^ mice with *EIIa-Cre* mice (stock no. 003314 from the Jackson Laboratory) to delete exons 5 and 6 and the *Neo* cassette flanked by the LoxP sequences. Heterozygous mice with *Sec23b* deletion (*Sec23b*^*ko/+*^) were continually crossed with C57BL/6J mice for at least five generations before analysis. Hepatocyte-specific mice were generated by crossing *Sec23b*^*fl/+*^ mice with *Alb-Cre* mice (stock no. 003574 from the Jackson Laboratory).

### Generation of SEC23B^E109K^ KI mice

To produce the *Sec23b*^E109K^ KI mice, 5′ homology arm (∼3.2 kb) and 3′ homology arm (∼7.2 kb) were cloned by PCR using C57BL/6 bacterial artificial chromosome DNA as a template. The E109K mutation was introduced into exon 4 (in the 5′ arm) by site-directed mutagenesis. In the targeting vector, the *Neo* cassette was flanked by LoxP sites. Homologous recombination was performed on C57BL/6 ES cells to identify the targeted allele. The targeted allele was identified by Southern blot. The *Neo* cassette was removed by transfection of the targeted clones with a *Cre*-expressing plasmid. Mice with germline transmission of the targeting allele were continually crossed with C57BL/6J mice for at least five generations before analysis.

### Mouse genotyping

Genotyping of *Sec23b* KI mice was carried out by a two-primer PCR assay of genomic DNA prepared from tail clippings of pups with primers CCF19 NeoF and CCF19 NeoR. A three-primer PCR assay was used to genotype *Sec23b* KO mice with primers: CCF13 DelR, CCF13 LoxPR, and CCF13 LoxPF. Primer sequences are listed in Table S1.

### Animal procedures

Blood glucose levels were measured using the Contour Blood Glucose Monitoring System (Bayer). The complete blood count was determined in an Advia120 whole blood analyzer (Bayer). Bone marrow was flushed from femurs and tibias of each mouse with Hanks' balanced salt solution. Bone marrow cytospin smear was stained by Wright's staining. GTTs were performed on 4-month-old mice after overnight fasting. The mice were injected intraperitoneally with a d-(+)-glucose bolus (2 g/kg of body weight). A small incision was made at the distal end of the tail vein, and blood glucose levels were measured before and at 15, 30, 60, and 120 min after glucose injection. Animals were refed immediately after the test.

### Measurement of total protein and proteases in mouse feces

Total proteases in fresh mouse feces were measured as previously reported (56). To measure total fecal protein, 200 mg of dried feces were homogenized in 3 ml water and incubated at 4 °C overnight. Samples were then centrifuged at 15,000*g* at 4 °C for 20 min. The supernatant was collected and stored at 4 °C. Next, 2 ml of 0.1 N NaOH was added to the pellet, and the mixture was gently rocked at room temperature for 1 h before centrifugation at 15,000*g* at 4 °C for 20 min. This supernatant was combined with the first supernatant. About 25% of trichloroacetic acid (TCA) was added to the combined supernatant with the ratio of 2.5:1. The sample was kept in ice for 30 min and centrifuged at 15,000*g* for 15 min at 4 °C. The supernatant was discarded, and the pellet was rinsed with cold 10% TCA followed by 5% TCA. The pellet was solubilized with 1 N NaOH. Protein concentrations were determined by the Bradford assay.

### RBC ghost preparation

One hundred microliters of peripheral blood was centrifuged at 3000 rpm in a microfuge. The pellet was washed twice with PBS and then lysed by suspension in ghost lysis buffer (5 mM Na_2_PO_4_, 1.3 mM EDTA; pH 7.6) containing protease inhibitors. Lysates were centrifuged at 16,000*g*, and the pellets containing the RBC membrane fraction were collected and washed five times in ghost lysis buffer. RBC ghosts were analyzed by SDS-PAGE, and proteins were visualized by Coomassie blue staining.

### Antibodies

Rabbit polyclonal anti-SEC23A and anti-SEC23B antibodies were purchased from MilliporeSigma (Burlington; ABC424 and ABC460). Rabbit polyclonal antibody against both paralogs of SEC23 (anti-COPII, PA1-069A) was purchased from Thermo Fisher Scientific. Anti-GHR antibody was purchased from Santa Cruz (sc-137185), and anti-β-actin was purchased from Sigma Aldridge (A5441). P-STAT5a/b, T-STAT5 a/b, P/T-AKT, and P/T extracellular signal–regulated kinase 1/2 were all purchased from Cell Signaling.

### Histological, Masson trichrome staining, and TUNEL staining

Tissues were fixed in 10% neutral buffered formalin solution (Fisher Scientific), embedded in paraffin, and cut into 5 μm–thick sections before H&E staining. Images were visualized and captured with a Zeiss Axioplan2 imaging microscope. Masson trichrome staining kit was purchased from Sigma Aldridge and performed according to the provided protocol. In cell death experiments, apoptotic cells in formalin-fixed and paraffin-embedded sections were detected by the TUNEL assay using a fluorescein-based detection kit (*in situ* death detection kit; Roche) according to the manufacturer's instructions. Sections were then examined under an inverted fluorescence microscope (Leica).

### TEM

Small pieces of pancreas were fixed in 2.5% glutaraldehyde and 4% formaldehyde for 24 h, followed by postfixation in 1% osmium tetroxide for 1 h. After en bloc staining and dehydration in a graded ethanol series, samples were embedded in eponate 12 medium (Ted Pella). Ultrathin sections (85 nm) were doubly stained with 2% uranyl acetate and 1% lead citrate and then observed using a PhilpsCM12 transmission electron microscope at an accelerating voltage of 60 kV by a person blind to the genotypes.

### RNA preparation and real-time RT–PCR

Total RNA was extracted using the Trizol reagent (Thermo Fisher Scientific) followed by purification using the RNA Mini kit (Thermo Fisher Scientific). RNA quantity and purity were determined by a Nanodrop Spectrophotometer. The total RNA (1 μg) from each sample was reverse transcribed into complementary DNA using the iScript cDNA select Synthesis Kit (Bio-Rad), according to the manufacturer's instructions. SYBR green–based quantitative PCRs were performed in a Bio-Rad CFX96 Real-Time PCR Detection System. Reaction specificity was determined by product melting curves. Relative gene expression was calculated by the 2^−ΔΔCt^ method using *Gapdh* as a reference gene. Primer sequences are listed in Table S1.

### Plasmids, mutagenesis, and cell-line transduction

A retroviral plasmid (pMSCV) expressing C-terminally GFP-tagged wildtype SEC23B was previously reported (25). Wildtype plasmids were mutagenized for the missense mutations of E109K and R14W with the QuikChange II Site-Directed Mutagenesis Kit (Agilent Technologies). All expression constructs were validated by Sanger sequencing prior to transduction. To generate Nthy-ori 3-1 stable cell lines, we kept retrovirally transduced cells under 1 mg/ml puromycin selection for >30 days prior to downstream experiments.

### Immunofluorescence staining

For Nthy-ori 3-1 immunofluorescence staining, cells were seeded on coverslips and fixed with 4% paraformaldehyde for 10 min at room temperature. Coverslips were blocked in 5% bovines serum albumin, permeabilized in 0.3% Triton X-100, and then incubated overnight with the primary antibodies. Fluorescent secondary antibodies conjugated with Alexa 488 or Alexa 594 (Thermo Fisher Scientific) were used for signal detection. Cellular nuclei were counterstained with 4′,6-diamidino-2-phenylindole. Coverslips were visualized, and images were obtained with a TCS SP5 confocal microscope (Leica).

### Immunoblotting

Immunoblotting protocol was described recently (57, 58). Blots were probed with either IRDye Infrared Fluorescent Dye conjugated secondary antibodies (LI-COR Biosciences) or horseradish peroxide–conjugated secondary antibodies. Images of infrared signals from IRDye-labeled antibodies were acquired using the Odyssey Infrared Imaging System (LI-COR Biosciences) and quantified using the Odyssey Image Studio. Images of chemiluminescent blot signals from horseradish peroxidase–conjugated secondary antibodies (Bio-Rad) were acquired by exposure to X-ray films or through the Amersham Imager 600 (GE Healthcare) and quantified using the ImageJ software (National Institutes of Health).

### Serum GH, IGF-1, thyroxine, amylase, and lipase levels

ELISA was performed to detect serum GH (rat/mouse GH ELISA kit; Merck KGaA), IGF-1 (mouse/rat IGF-1 ELISA kit; R&D Systems), and total thyroxine (total thyroxine ELISA; ALPCO) according to the protocols of the respective ELISA kits. Serum amylase and lipase activities were measured using kits from BioAssay Systems (ECAM-100 and DLPS-100) according to the manufacturer's instructions.

### Statistical analysis

All data are presented as mean ± SD. ANOVA with Tukey post hoc test or two-tailed Student's *t* test was used for comparison of continuous variables, as appropriate, and Chi-squared test was used for comparison of binary variables. All *p* values were two-sided, and *p*  <  0.05 was considered statistically significant.

### Study approval

Animal experimental protocols were approved by the Institutional Animal Care and Use Committee of Cleveland Clinic Lerner Research Institute.

## Data availability

The data generated are included in the main text file and supporting information.

## Supporting information

This article contains supporting information (25, 26).

## Conflict of interest

The authors declare that they have no conflicts of interest with the contents of this article.
