# Supplementary material for: A common human missense mutation of vesicle coat protein SEC23B leads to growth restriction and chronic pancreatitis in mice
Source: J Biol Chem. 2021 Dec 24;298(1):101536. doi: 10.1016/j.jbc.2021.101536 (PMC8760524; doi:10.1016/j.jbc.2021.101536)
Supplement: Supporting information [file mmc1.pdf]

A common human missense mutation of vesicle coat protein SEC23B leads to growth restriction and chronic pancreatitis in mice

Wei Wei[1], Zhigang Liu[1], Chao Zhang[2], Rami Khoriaty[3], Min Zhu[4], Bin Zhang[1, ¶]

Supplemental methods

Cell culture and transfection. The HEK293 cell line was cultured in DMEM supplemented with 2 mM glutamine and 10% FBS. All cell lines were maintained at 37 ºC and 5% CO2 culture conditions and tested negative upon routine mycoplasma testing with the MycoAlert Mycoplasma Detection Kit (Lonza) at the C.E. lab (luminescence ratios < 0.9). GFP-tagged wild-type and mutant SEC23B cloned into the pMSCV plasmid (1) were transfected into HEK293 cells using FuGene 6 (Promega). Cells were analyzed 48 h post transfection.

Immunofluorescence staining. For immunofluorescence analysis of insulin and glucagon (2), pancreatic tissues were fixed in 4% paraformaldehyde, washed and incubated in 30% sucrose, before cryo-embedding. Sagittal, transverse and coronal 5-μm-thick sections or fixed pancreas were blocked in 5% BSA, permeabilized in 0.3% Triton X-100, and then incubated overnight with the primary antibodies. Fluorescent secondary antibodies conjugated with Alexa 488 or Alexa 594 was used for signal detection. Cellular nuclei were counterstained with 4,6-diamidino-2-phenylindole (Dapi). Sections were then examined under an inverted fluorescence microscope (Leica).

Characterization of E18.5 Sec23b$^{ko/ko}$ embryos. Time mating of *Sec23b*$^{ko/+}$ X *Sec23b*$^{ko/+}$ cross, dissection of pancreas from E18.5 embryos, and H&E staining of pancreas cryosections were performed as previously reported (2).

Measurements of pro-inflammatory cytokines. Serum TNFα, IL-1 and IL-6 levels were measured using ELISA kits (R&D Systems) according to manufacturer instructions.

References

1. Yehia L, Niazi F, Ni Y, Ngeow J, Sankunny M, Liu Z, et al. Germline Heterozygous Variants in SEC23B Are Associated with Cowden Syndrome and Enriched in Apparently Sporadic Thyroid Cancer. *Am J Hum Genet.* 2015.
2. Tao J, Zhu M, Wang H, Afelik S, Vasievich MP, Chen XW, et al. SEC23B is required for the maintenance of murine professional secretory tissues. *Proc Natl Acad Sci U S A.* 2012;109(29):E2001-E9.

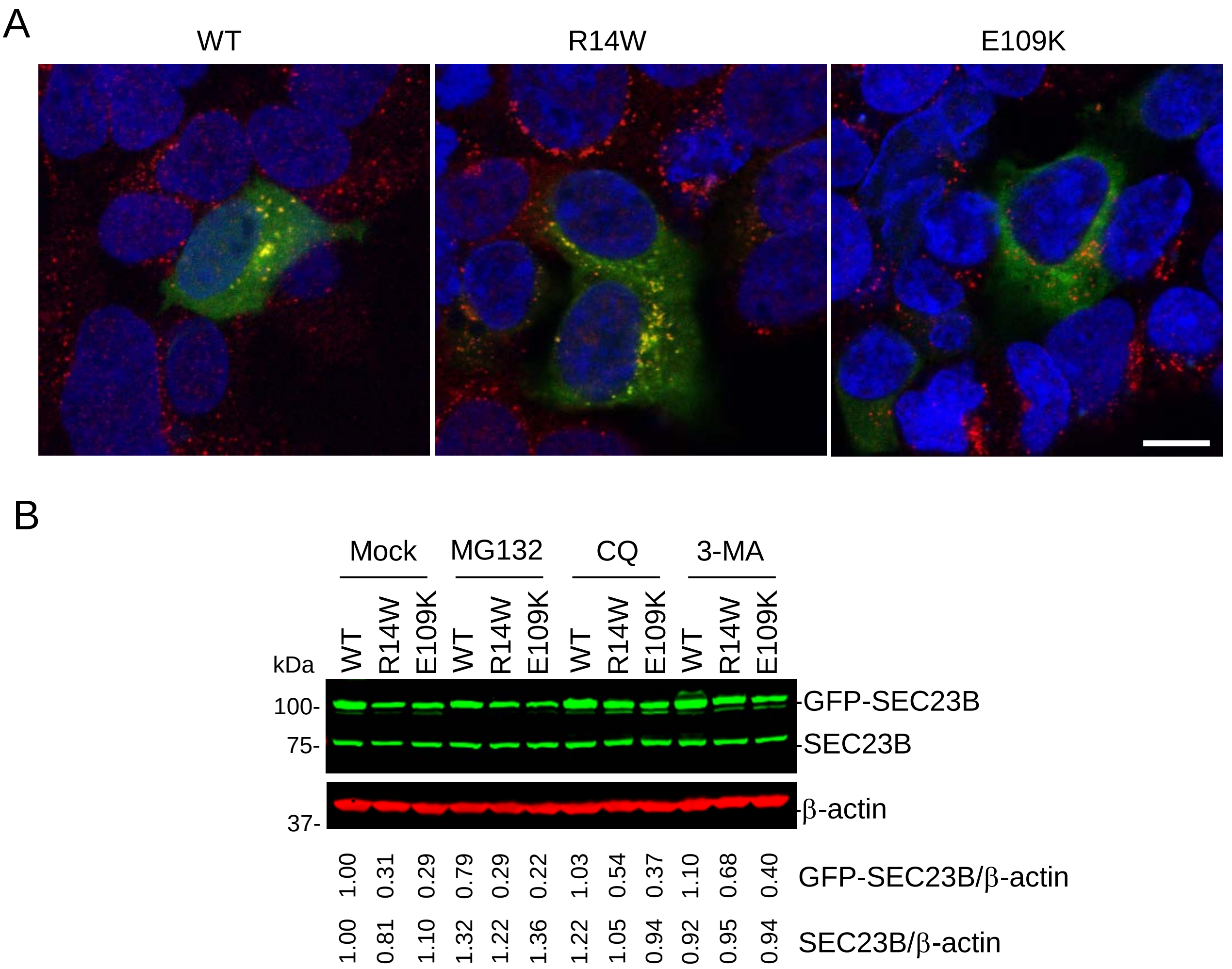

Fig. S1. Analysis of SEC23B mutants by transient transfection. (A) Immunofluorescence staining of HEK293 cells transiently transfected with plasmids expressing WT, R14W and E109K SEC23B-GFP fusion proteins. Cells were stained with rabbit anti-SEC16A (red) for ER exit sites and DAPI (blue) for nuclei. Exogenous SEC23B-GFP fusion protein was shown in green. Scale bar: 10 µm. (B) HEK293 cells expressing GFP-SEC23B fusion proteins were treated with MG132, chloroquine (CQ) and 3-methyladenine (3-MA) for 24 hours. Immunoblotting analysis was performed using IRDye conjugated antibodies and band intensity ratios of GFP-SEC23B/β-actin and endogenous GFP-SEC23B/β-actin are indicated under the gel images. Experiments were repeated 3 times.

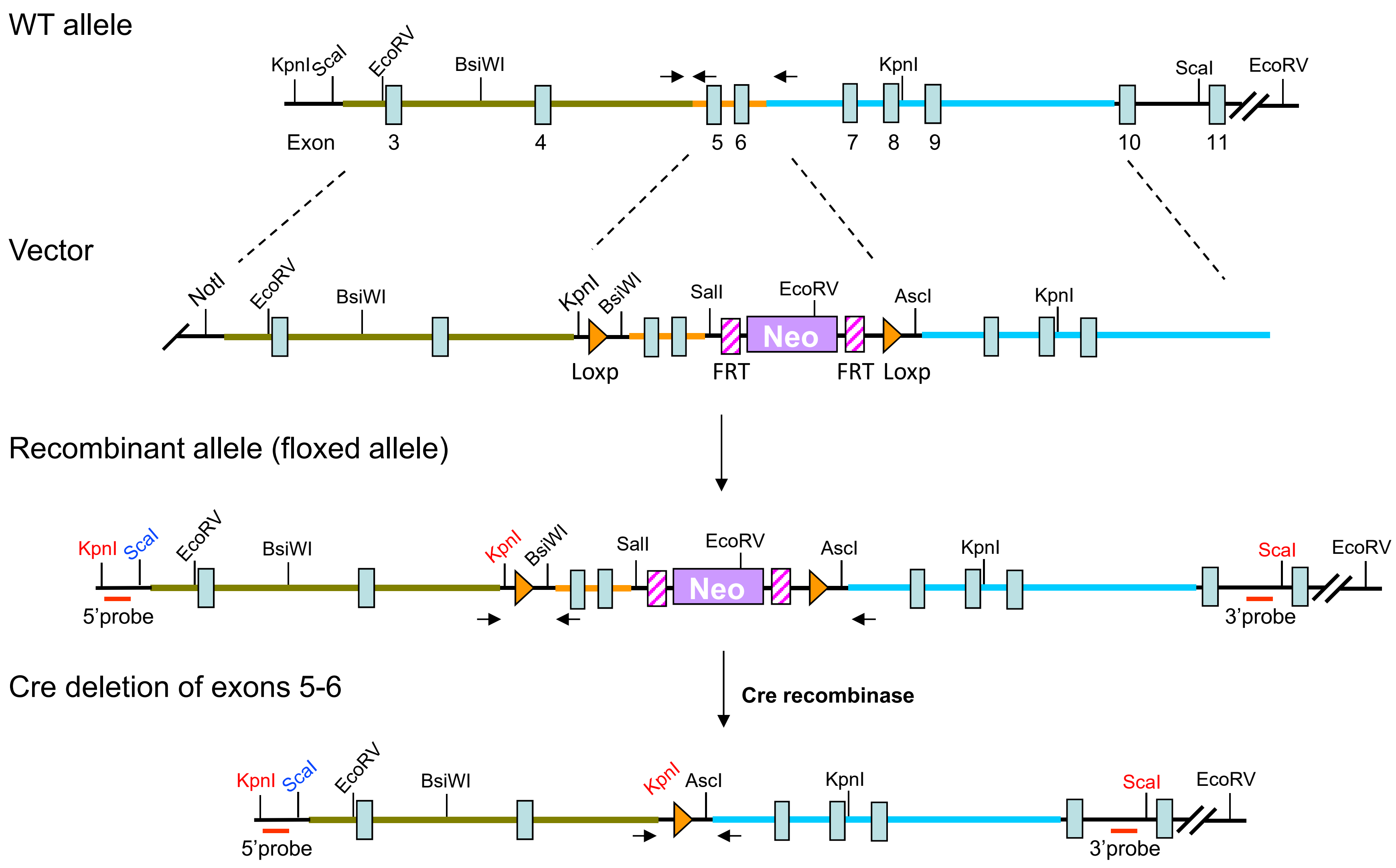

Figure S2. Detailed diagram of *Sec23b* conditional knockout mouse generation. Arrows denote locations of genotyping primers. Short red bars denote locations of Southern blot probes.

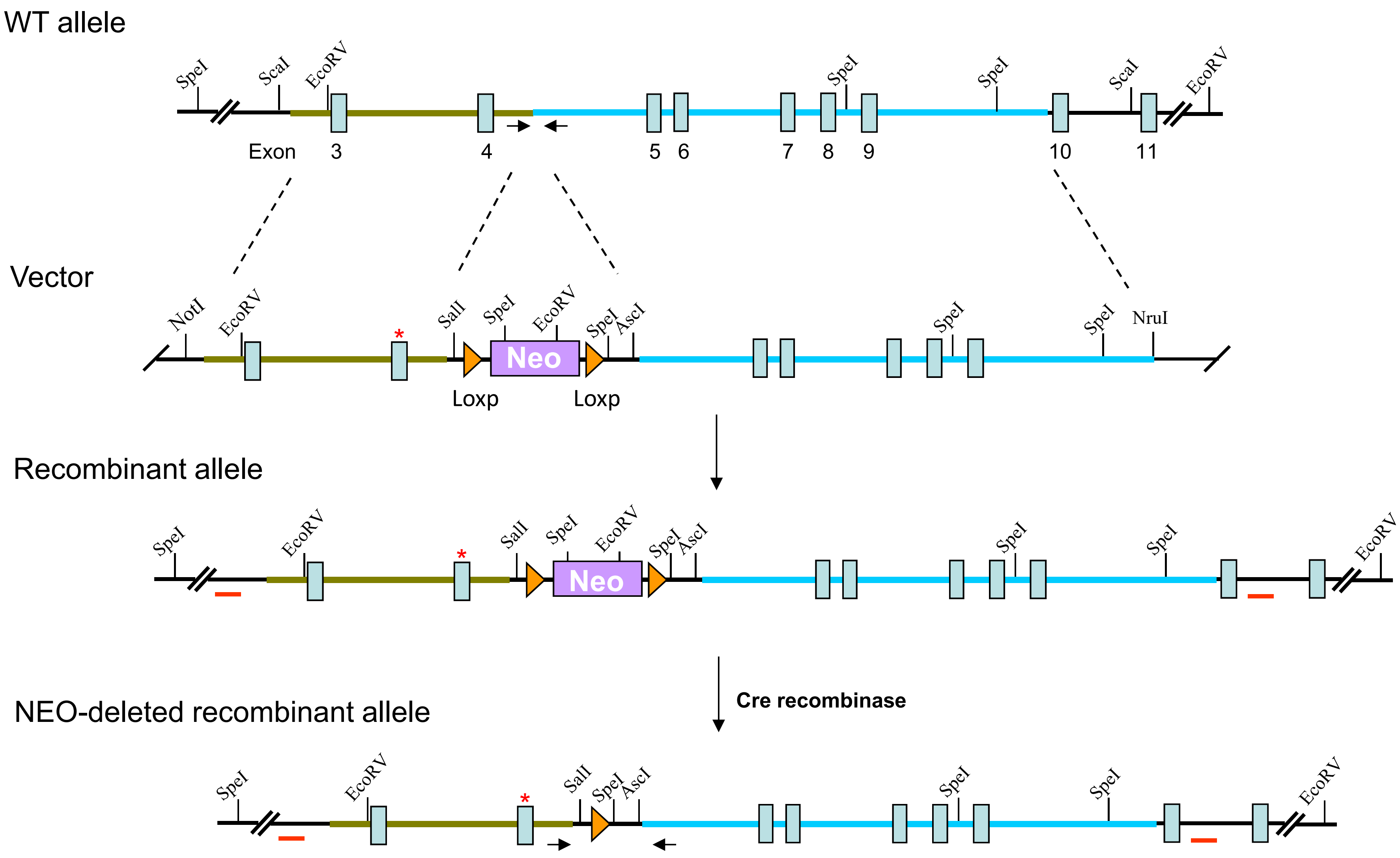

Figure S3. Detailed diagram of *Sec23b^{E109K}* knockin mouse generation. Asterisks indicate the location of E109K mutation. Arrows denote locations of genotyping primers. Short red bars denote locations of Southern blot probes.

A

Genotype distribution of pups at weaning from intercrosses of *Sec23b*$^{ko/+}$ mice

	Genotype			P value
	+/+ (n)	ko/+ (n)	ko/ko (n)	
Expected ratio	25%	50%	25%	
P21 observed ratio (n=38)	31.6% (12)	68.4% (26)	0% (0)	<0.001

B

WT *Sec23b*$^{ko/ko}$

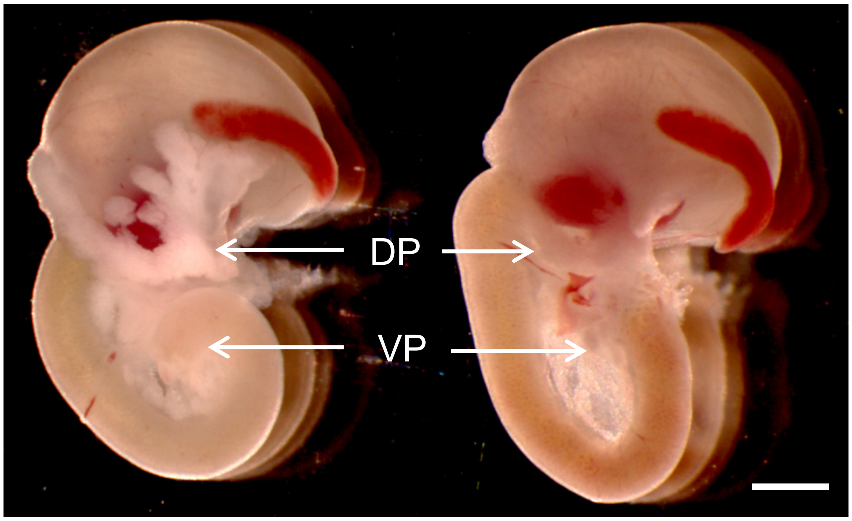

C

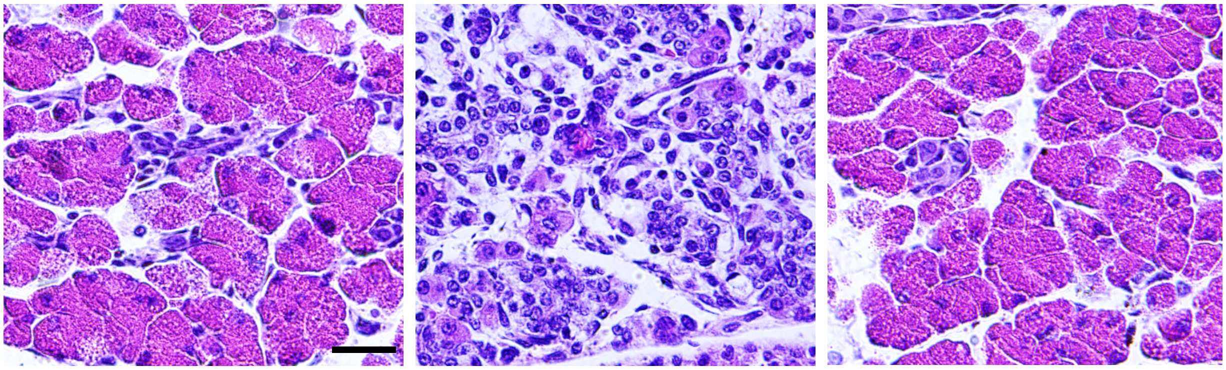

WT *Sec23b*$^{ko/ko}$ *Sec23b*$^{ki/ko}$

Figure S4. Pancreas defects in E18.5 *Sec23b*$^{ko/ko}$ embryos. (A) Absence of *Sec23b*$^{ko/ko}$ mice and no loss of *Sec23b*$^{ko/+}$ embryos at weaning. (B) Pancreatic tissues dissected from E18.5 embryos are smaller and less opaque than from WT and *Sec23b*$^{ko/+}$ embryos. DP, dorsal pancreas; VP; ventral pancreas. Scale bar: 1 mm. (C) H&E staining of cryosections of E18.5 pancreatic tissues demonstrates extensive destruction of pancreatic parenchyma and the lack of zymogen contents in *Sec23b*$^{ko/ko}$ pancreas compared to WT and *Sec23b*$^{ko/+}$ pancreas. Scale bar: 50 µm.

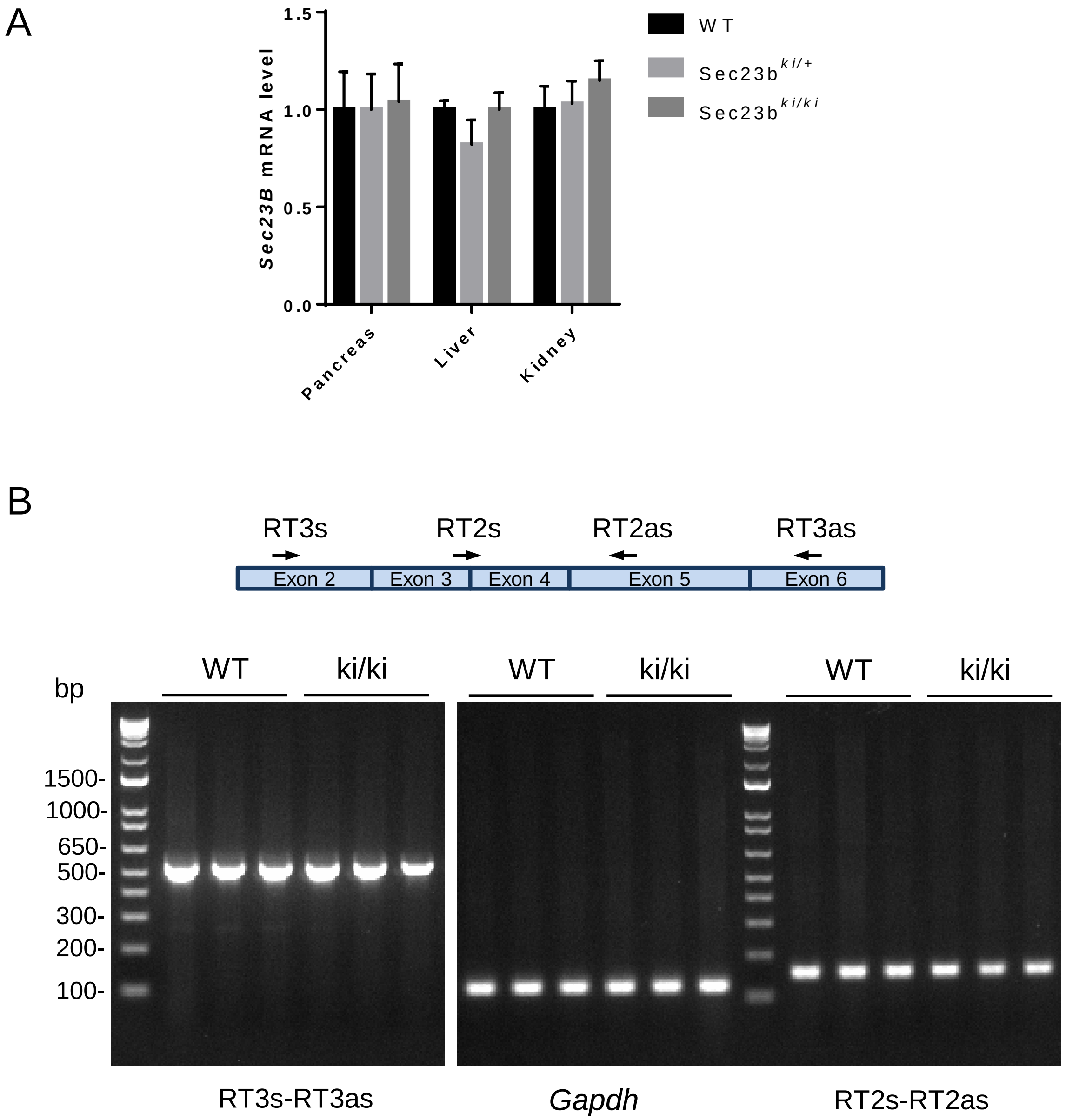

Figure S5. The remnant loxP sequence does not affect transcription and splicing of *Sec23b* mRNA. (A) Real time RT-PCR analysis of *Sec23b* mRNA from pancreas, liver and kidney of WT, *Sec23b*$^{ki/+}$ and *Sec23b*$^{ki/ki}$ mice (n=7 for each genotype) using primers RT1s and RT1as (26). (B) RT-PCR analysis using primers flanking the exon 4 and exon 5 junction revealed no alternative splicing of pancreas mRNA in *Sec23b*$^{ki/ki}$ mice. Each lane represents sample from a single mouse. Primer sequences are listed in Table S1.

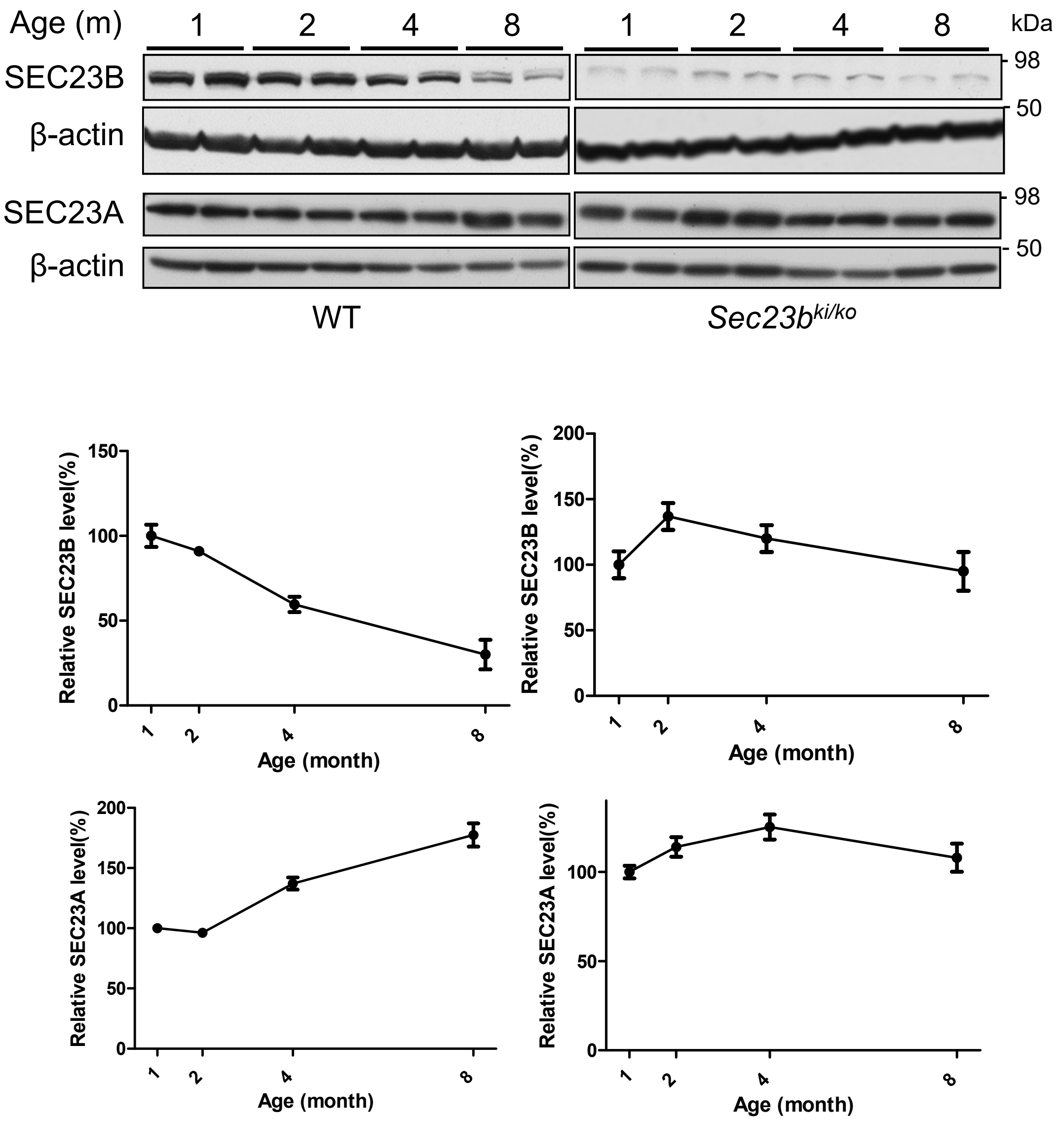

Figure S6. Changes in relative SEC23 paralog levels in pancreas of WT and *Sec23b*$^{ki/ko}$ mice. SEC23B, SEC23A and β-actin in pancreas were detected by immunoblotting from WT and *Sec23b*$^{ki/ko}$ mice of the indicated ages. Two mice of each genotype were analyzed for each time point. Densitometric values of SEC23B and SEC23A bands relative to β-actin bands were plotted.

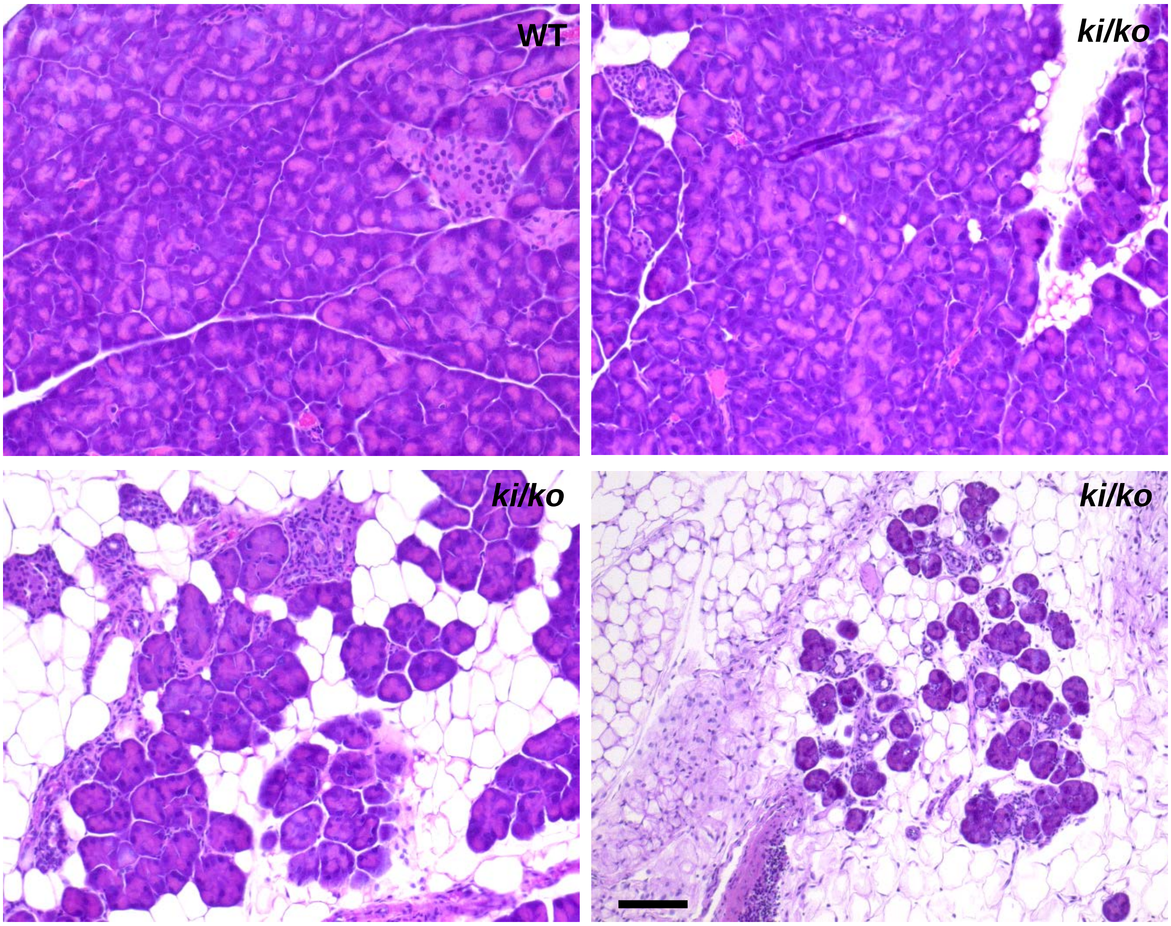

Figure S7. Ranges of abnormal pancreas morphology of *Sec23b*$^{ki/ko}$ mice. Representative H&E staining of pancreas from WT was shown at upper left panel. Images of H&E staining of pancreas from *Sec23b*$^{ki/ko}$ *(ki/ko)* mice were chosen to show different stages of exocrine destruction. Note that relative intact structures of islets persist after surrounding acinar cells have been replaced by fat cells, suggesting that acinar destruction is the primary defect. Scale bar: 50 µm.

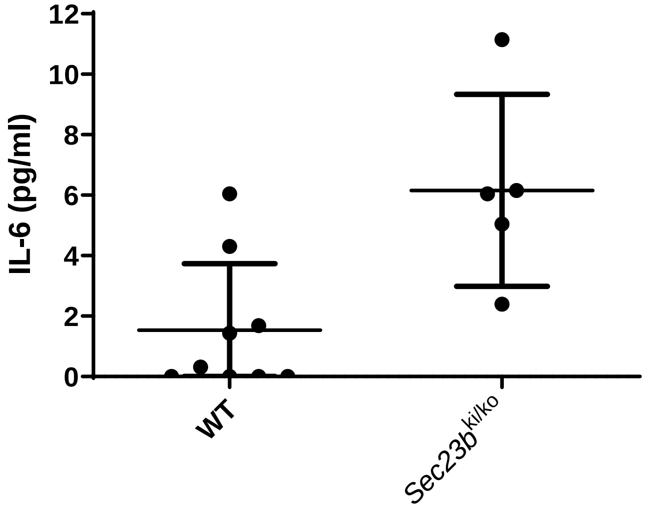

Figure S8. Comparison of serum IL-6 levels between WT and *Sec23b*$^{ki/ko}$ mice. Serum was collected form 2-month old mice and analyzed by IL-6 ELISA. Error bars represent mean $\pm$ SD, P<0.01.

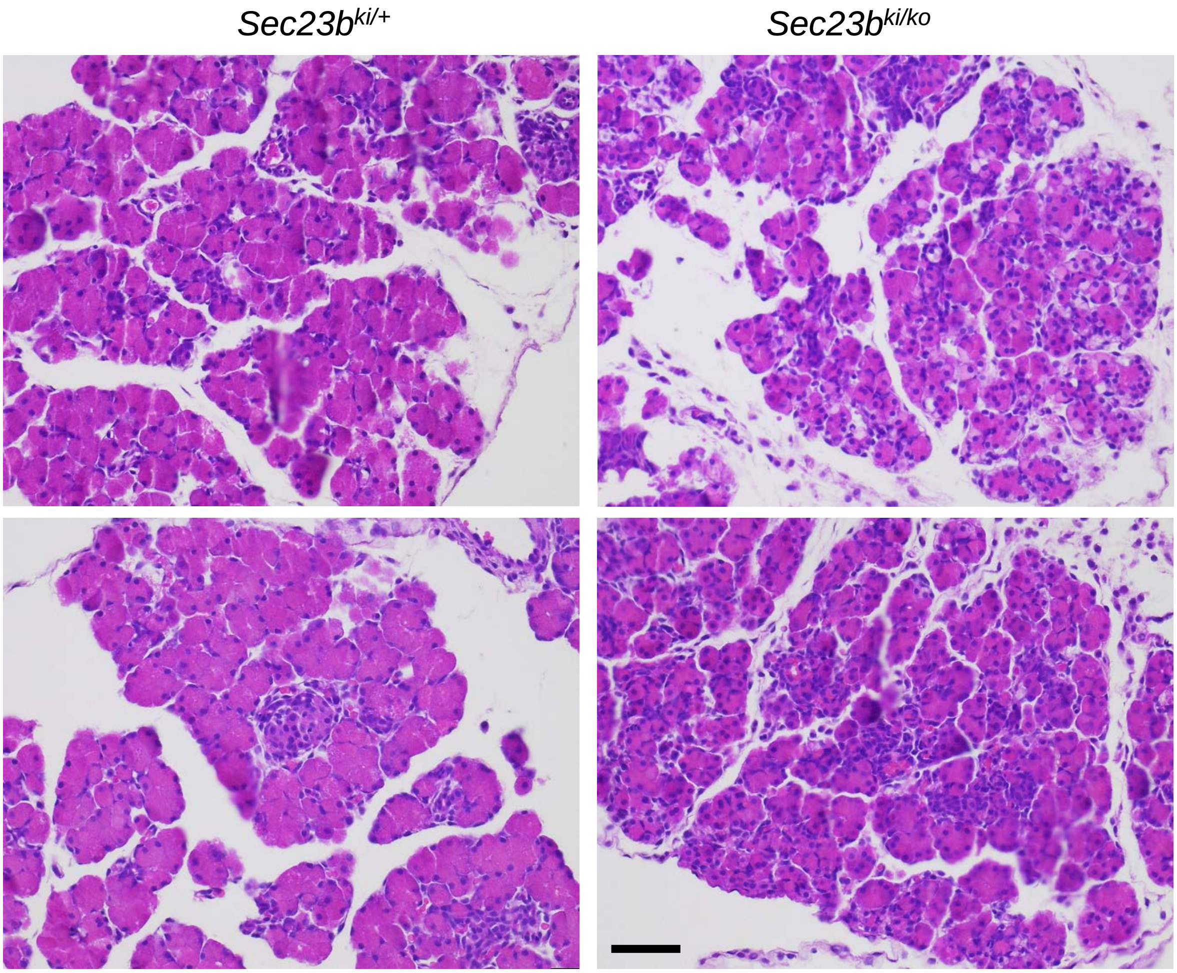

Figure S9. Pancreas defects in *Sec23b*$^{ki/ko}$ neonates. H&E staining of paraffin-embedded formalin-fixed P0 pancreatic tissues demonstrates signs of lymphocyte infiltration and minor degeneration of acinar cells in *Sec23b*$^{ki/ko}$ pancreas compared to *Sec23b*$^{ki/+}$ pancreas. Scale bar: 50 µm.

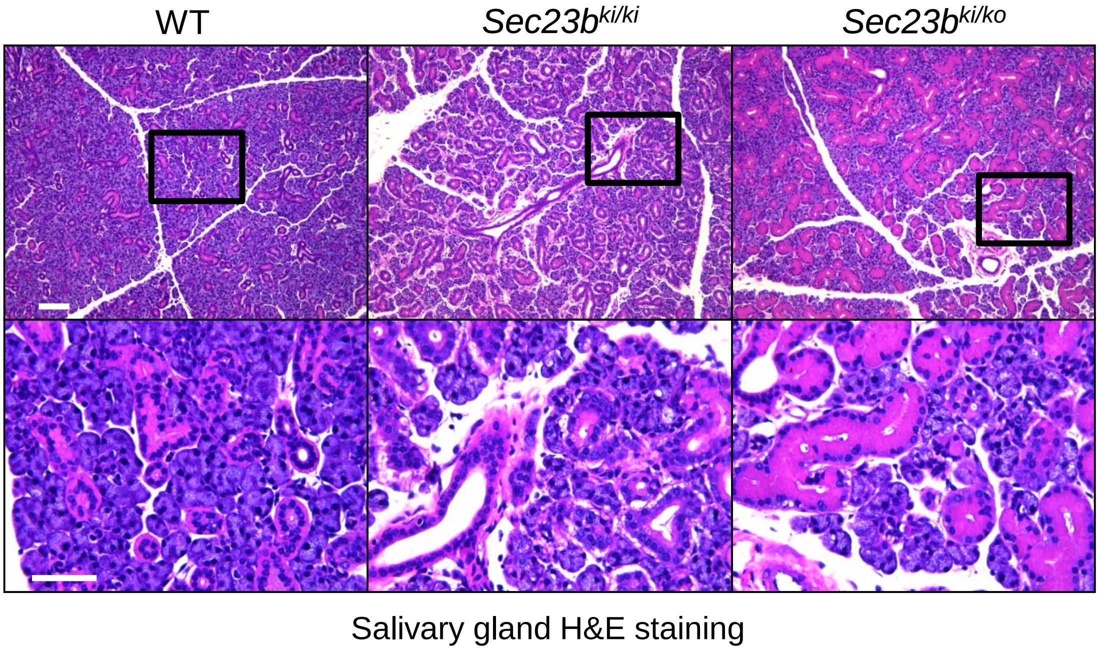

Figure S10. No obvious abnormalities in salivary gland tissue structures. Submandibular glands from WT, *Sec23b*$^{ki/ki}$ and *Sec23b*$^{ki/ko}$ mice were analyzed by H&E staining of paraffin-embedded formalin-fixed tissues. Scale bars: 100 µm (top) and 50 µm (bottom). Three 2-month old mice from each genotype were analyzed.

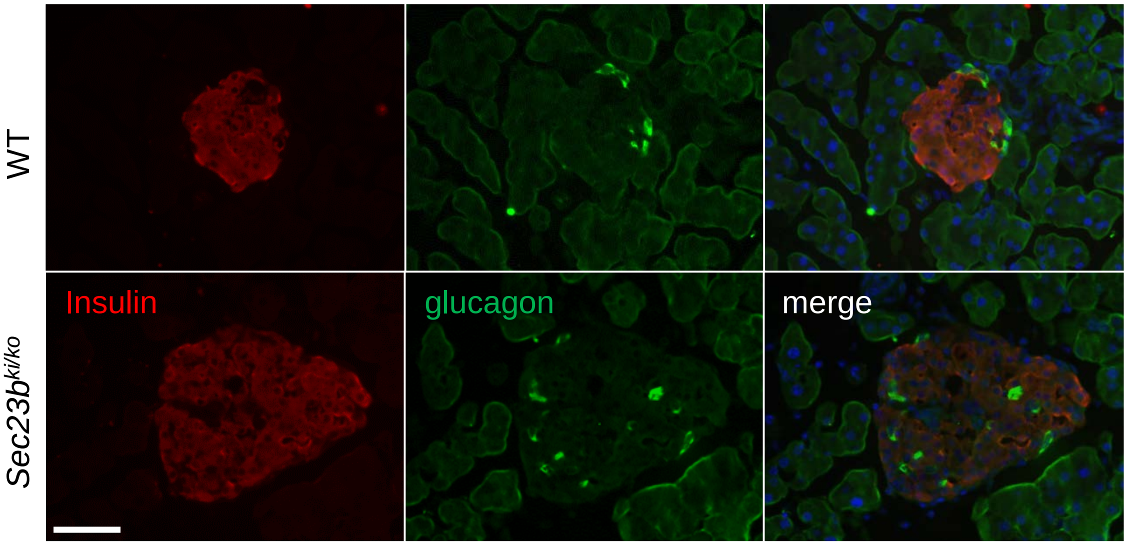

Figure S11. Minor islet structural defects in *Sec23b*$^{ki/ko}$ mice. Cryosections of pancreas from WT and *Sec23b*$^{ki/ko}$ mice of 4 month of age were co-stained with mouse anti-glucagon (green) and guinea pig anti-insulin (red) for α- and β-endocrine cells, respectively. Scale bar: 50 µm. Two mice from each genotype were analyzed.

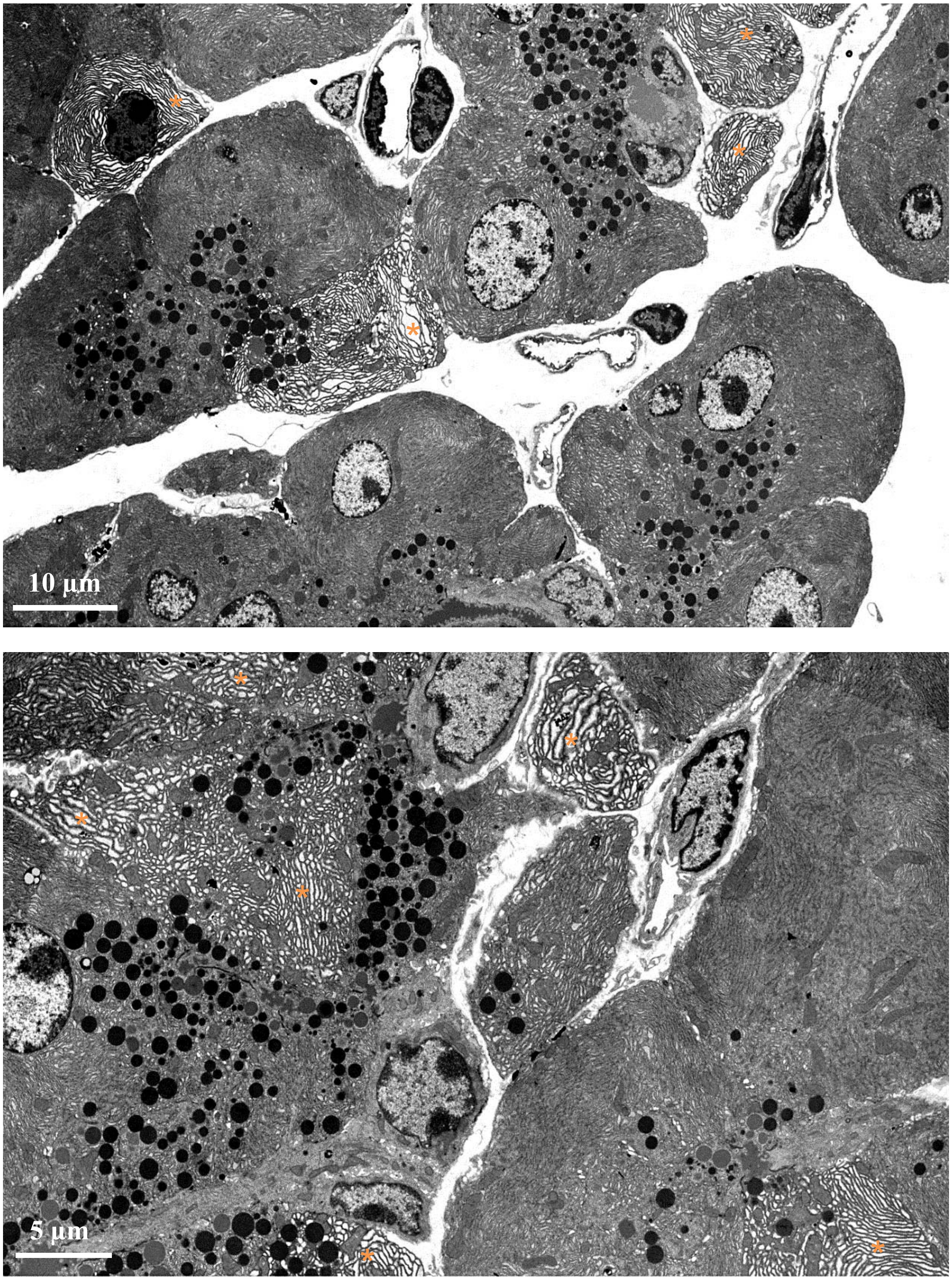

Figure S12. TEM image of representative groups of pancreatic acinar cells. TEM images were obtained from pancreas tissues of 2-month old *Sec23b*$^{ki/ko}$ mice and show acinar cells ranging from those with normal morphology to those at variant stages of ER stress and apoptosis. Orange asterisks denote likely apoptotic cells.
