# Supplementary material for: A common human missense mutation of vesicle coat protein SEC23B leads to growth restriction and chronic pancreatitis in mice
Source: J Biol Chem. 2021 Dec 24;298(1):101536. doi: 10.1016/j.jbc.2021.101536 (PMC8760524; doi:10.1016/j.jbc.2021.101536)
Supplement: Supplementary file 2 — Table S1 [file mmc2.pdf]

Table S1. Primers used in the study.

Primer name	Sequence (5' to 3')	Use
CCF19 NeoF	GGAGCACCAATCACTTTGAGCCC	KI mouse genotyping
CCF19 NeoR	AAGGGAGATCATGGGAAGGGTGG	KI mouse genotyping
CCF13 LoxPF	GATAGACTCTGGGTCCTCATCATTGGA	KO mouse genotyping
CCF13 LoxPR	GACATAACCAGCGAGCACAGAGAGAC	KO mouse genotyping
CCF13 delR	CAACAGCAATGGACAAAGCAACAC	KO mouse genotyping
Gapdh-S	AGGTCGGTGTGAACGGATTTG	qRT-PCR for Gapdh gene
Gapdh-AS	TGTAGACCATGTAGTTGAGGTCA	qRT-PCR for Gapdh gene
Xbp1-sF	GAGTCCGCAGCAGGTG	qRT-PCR for spliced Xbp1 gene
Xbp1-sR	GTGTCAGAGTCCATGGGA	qRT-PCR for spliced Xbp1 gene
Atf4-F	ATGGCCGGCTATGGATGAT	qRT-PCR for Atf4 gene
Atf4-R	CGAAGTCAAACTCTTTCAGATCCATT	qRT-PCR for Atf4 gene
Edem1-F	GCAATGAAGGAGAAGGAGACCC	qRT-PCR for Edem1 gene
Edem1-R	TAGAAGGCGTGTAGGCAGATGG	qRT-PCR for Edem1 gene
Gadd34-F	CCCGAGATTCCTCTAAAAGC	qRT-PCR for Ppp1r15a gene
Gadd34-R	CCAGACAGCAAGGAAATGG	qRT-PCR for Ppp1r15a gene
Grp78-F	CATGGTTCTCACTAAAATGAAAGG	qRT-PCR for Hspa5 gene
Grp78-R	GCTGGTACAGTAACAACTG	qRT-PCR for Hspa5 gene
Grp94-F	TCGTCAGAGCTGATGATGAAGT	qRT-PCR for Hsp90b1 gene
Grp94-R	GCGTTTAACCCATCCAACTGAAT	qRT-PCR for Hsp90b1 gene
Chop-F	CTGGAAGCCTGGTATGAGGAT	qRT-PCR for Ddit3 gene
Chop-R	CAGGGTCAAGAGTAGTGAAGGT	qRT-PCR for Ddit3 gene
Trb3-F	TCTCCTCCGCAAGGAACCT	qRT-PCR for Trib3 gene
Trb3-R	TCTCAACCAGGGATGCAAGAG	qRT-PCR for Trib3 gene
Ghr-F	ACAGTGCCTACTTTTGTGAGTC	qRT-PCR for Ghr gene
Ghr-R	GTAGTGGTAAGGCTTTCTGTGG	qRT-PCR for Ghr gene
Igf1-F	TCAGACAGGCATTGTGGATGAG	qRT-PCR for Igf1 gene
Igf1-R	GGACGGGGACTTCTGAGTCTT	qRT-PCR for Igf1 gene
Cebpb-F	ACCGGGTTTCGGGACTTGA	qRT-PCR for Cebpb gene
Cebpb-R	GTTGCGTCAGTCCCGTGTCCA	qRT-PCR for Cebpb gene
c-Fos-F	CGGGTTTCAACGCCGACTA	qRT-PCR for Fos gene
c-Fos-R	TTGGCACTAGAGACGGACAGA	qRT-PCR for Fos gene
Socs3-F	ATGGTCACCCACAGCAAGTTT	qRT-PCR for Socs3 gene
Socs3-R	TCCAGTAGAATCCGCTCTCCT	qRT-PCR for Socs3 gene
Sec23b RT2s	GAAATCAGTTCCCTCCAGCA	RT-PCR for Sec23b gene
Sec23b RT2as	TTCCTCCAAACACGTGTCAA	RT-PCR for Sec23b gene
Sec23b RT3s	AACGCCCAGACTTACCTCCT	RT-PCR for Sec23b gene
Sec23b RT3as	GAAACAAAAGGCTGCTCCTG	RT-PCR for Sec23b gene
